# The Disruption of the HIV-1 Gag Start Codon via Editing Using MmCas12m-Dual Base Editor-Loaded Virus-like Particles

**DOI:** 10.3390/cimb48030241

**Published:** 2026-02-25

**Authors:** Timur Aliev, Almaz Imatdinov, Elena Prudnikova, Oleg Taranov, Ksenia Emtsova, Ilnaz Imatdinov, Alexander Agafonov

**Affiliations:** 1 State Research Center of Virology and Biotechnology “Vector”, 630559 Kol’tsovo, Russia; imatdinov_ar@vector.nsc.ru (A.I.); prudnikova_eyu@vector.nsc.ru (E.P.); taranov@vector.nsc.ru (O.T.); emtsova_kf@vector.nsc.ru (K.E.); imatdinov_ir@vector.nsc.ru (I.I.); agafonov@vector.nsc.ru (A.A.); 2 Faculty of Natural Sciences, Novosibirsk State University, 630090 Novosibirsk, Russia

**Keywords:** HIV, gene therapy, base editing, CRISPR/Cas, MmCas12m, virus-like particles

## Abstract

Approaches to delivering gene editing tools in the form of ribonucleoproteins may provide a safety advantage over the delivery of nucleic acids encoding ribonucleoproteins. Virus-based vectors are widely used as a delivery platform. However, the persistence of viral exogenous nucleic acids can cause increased genotoxicity. Virus-like particles (VLPs) do not contain an expression cassette and can act as a platform for the delivery of ready-made ribonucleoprotein complexes. The absence of nucleic acids in VLPs eliminates the risk of insertional mutagenesis compared to widely used lentiviruses or adeno-associated viruses. Therefore, we used VLPs to deliver the ribonucleoprotein complex MmCas12m–TadDE to disrupt the HIV-1 *gag* gene start codon. We detected VLP morphogenesis using electron microscopy. We confirmed the incorporation of MmCas12m–TadDE into VLPs. We achieved an editing efficiency of about 9% in some cases with minimal off-target effects, which confirms the prospect of using VLPs as a platform for delivering genomic editing tools.

## 1. Introduction

Virus-like particles (VLPs) are structures composed of assembled viral proteins that do not contain any viral genetic materials. For this reason, VLPs are non-infectious, although VLPs simulate a form and size of the virus particles on the basis of how they were obtained [1]. The production of VLPs, as well as other viral vectors, can be carried out in the producer cells, for example, HEK293T [2]. In terms of lentivirus-based VLPs, their formation in the producer cells occurs as a result of the spontaneous assembly of polyproteins that encapsulate protein molecules of interest, with further budding of the virion from the host cell plasma membrane [3]. VLPs do not contain a viral genome, but at the same time retain the ability to deliver the protein cargo to cells. Thus, VLPs can act as a platform for the delivery of various protein structures [4,5]. VLPs based on various viruses have successfully proven themselves for the delivery of various variants of the Cas9 nuclease [6,7,8,9,10]. VLPs were also used for the efficient delivery of base editors [11]. VLPs are based on various viruses, including lentivirus, mouse leukemia virus, adeno-associated virus (AAV), and hepatitis B virus [12,13,14].

VLPs have some advantages over other viral delivery platforms. VLPs do not have strict restrictions on the size of the protein cargo, because VLPs do not deliver expression cassettes, but pre-synthesized complexes. Thus, the delivery of voluminous genomic editing tools, for example, prime editing, can be delivered by a single VLP. The absence of a viral genome excludes insertional mutagenesis. Furthermore, the frequency of off-target editing is significantly reduced by minimizing the persistence of the genome editing tool in the cells. In addition, VLPs can be pseudotyped by various glycoproteins for tissue-specific interactions [15,16,17,18]. Therefore, VLPs have already demonstrated some success in the field of gene therapy of hereditary human diseases [19].

The main goal of our research is the disruption of the *gag* start codon using the new MmCas12m–TadDE base editor. We used virus-like particles to deliver the new editing tool. The MmCas12m variant isolated from *Mycolicibacterium mucogenicum* may be useful in genetic engineering applications, as it has a number of differences from the widely used Cas9 proteins. Even the wild-type lacks the catalytic activity. The MmCas12m variant has a compact size (596 amino acids) compared to other Cas9 protein variants. Thus, the MmCas12m can be easily delivered using a single AAV-based vector. And its small size is also advantageous for VLPs’ packaging efficiency. Furthermore, MmCas12m has a greater binding strength to the target DNA than other variants of Cas proteins. This is explained by the fact that the native function of MmCas12m is to suppress transcription from exogenous nucleic acids solely by binding to the DNA without introducing double-stranded breaks [20,21]. Therefore, MmCas12m can act as a transcription inhibitor without any modifications or fused effector proteins, for example, to prevent viral replication or viral transcription. Additionally, we have fused MmCas12m with the new recombinant dual deaminase based on TadA-8e (TadDE). TadDE has the ability to deaminate adenine and cytosine according to the original study [22]. The dual base editor can be useful in cases of multiple mutagenesis. The start codon region of the HIV-1 *gag* gene was selected as a target for the MmCas12m-TadDE ribonucleoprotein complex, carried by VLPs. Disruption of the *gag* start codon or inhibition of transcription from the proviral DNA has a therapeutic potential and can be considered as one of the possible ways to solve the problem of HIV gene therapy. Targeting the *gag* start codon is a robust way to prevent viral assembly, even if the provirus remains.

## 2. Materials and Methods

### 2.1. Cells

Human embryonic kidney 293 T (HEK293T, Cat. no. 273) cells and TZM-bl cells (modified HeLa cell line, expressing luciferase and β-galactosidase reporter genes under the control of the HIV-1 long terminal repeat, Cat. no. 307) were obtained from the cell culture collection of the Federal Budgetary Institution of Science, “State Research Center of Virology and Biotechnology ‘Vector’”, Russia. Cells were grown in Dulbecco’s modified Eagle’s medium F12 (DMEM/F12, BioloT, Moscow, Russia). TZM-bl cells (also called JC53BL-13) were obtained previously from the NIH AIDS Research and Reference Reagent Program (Original cat. no. 8129, Durham, NC, USA). The growth medium contained an additional 10% heat-inactivated fetal bovine serum, FBS (BioloT), and penicillin–streptomycin (BioloT). The cells were cultured at 37 °C in a humidified atmosphere of 5% CO_2_.

### 2.2. Plasmids

The following plasmids were used for the study (Table 1).

Restriction endonucleases and DNA ligases used for cloning are from NEB (New England Biolabs, Orlando, FL, USA). All oligonucleotides were synthesized by Evrogen (Evrogen, Moscow, Russia). Nucleotide sequences of plasmids (Table 1) were confirmed by Sanger sequencing using a BigDye Terminator v3.1 Cycle Sequencing kit (Thermo Fisher Scientific, Waltham, MA, USA) on an ABI 3500/3500xl Genetic Analyzer (Applied Biosystems, Carlsbad, CA, USA). Plasmid DNA was purified using a Plasmid Miniprep kit (#T1010L, New England Biolabs). DNA fragments from agarose gel were purified using a Cleanup Mini (#BC023L, Evrogen). If necessary, the authors can provide details of cloning, for example, annealing temperature for PCR, elongation time, the DNA ligation protocol, etc.

pMmCas12m-sgRNA and pdSpCas9-sgRNA plasmids were based on the transfer plasmid pLenti CMV GFP Puro (#17448, Addgene). The GFP reporter gene, the CMV promoter and the CMV enhancer were removed from the original plasmid. Instead, the MmCas12m (synthesized de novo) or catalytically inactive SpCas9 (dSpCas9) (a donor plasmid is #201953, Addgene) was cloned under the control of the PGK promoter. Each Cas protein was flanked by two nuclear localization signals (NLSs). Additionally, the specific tracrRNA sequence of MmCas12m and SpCas9 was cloned into pMmCas12m-sgRNA and pdSpCas9-sgRNA under control of the U6 promoter, respectively. Two BsaI sites are located after the tracrRNA sequence for cloning any crRNA sequence. During transcription, synthesized sgRNAs molecules consist of tracrRNA and crRNA parts. sgRNAs oligonucleotides were ligated into pMmCas12m-sgRNA or pdSpCas9-sgRNA plasmids via BsaI sites. Thus, a set of pMmCas12m-sgRNA*N* and pdSpCas9-sgRNA*N* plasmids was obtained (N is the number of sgRNA).

pMmCas12m-TadDE-sgRNA and pdSpCas9-TadDE-sgRNA plasmids are based on pMmCas12m-sgRNA and pdSpCas9-sgRNA with some modifications. In particular, the GFP reporter gene was cloned before each Cas gene, and the TadDE gene was cloned after the Cas gene. GFP is necessary for transfection visualization. Additionally, the P2A sequence is localized between the GFP and a Cas gene. The self-cleaving P2A peptide is needed to separate the GFP from the Cas-TadDE editing complex. Cas and TadDE are fused by a flexible glycine–serine linker for the mobility of the domains relative to each other. The full nucleotide sequence of both editing complexes is shown in Supplementary Figures S1 and S2.

A set of plasmids was obtained for VLP production. pGag-MmCas12m-TadDE and pGag-dSpCas9-TadDE encode engineered HIV-1 Gag-Cas-TadDE-6×His fusion proteins under the control of the CMV promoter. The psPAX2 plasmid was used as a basis. The nucleotide sequence of both fusion proteins (Supplementary Figures S3 and S4) was cloned into psPAX2 via SphI and XcmI sites instead of the original sequence. psgRNA-MmCas12m and psgRNA-dSpCas9 encode specific single-guide RNA under the control of the U6 promoter. psgRNA-MmCas12m and psgRNA-dSpCas9 were obtained based on a donor plasmid (#122089, Addgene) by replacing the original sequence with the target sequence (Supplementary Figure S5) via PciI and KpnI sites.

pKW was created by converting the Kozak sequence from GCCACCAUG to GATATCAUG in phMGFP using specific primers for site-directed mutagenesis. Note that GATATC is an EcoRV restriction site and can be used for cloning. The phMGFP plasmid codes GFP under the control of the CMV promoter. The Psi and the *gag* gene fragments were cloned in pKW upstream of the reporter gene in-frame with GFP (pKW_R1) and with reading frameshifts of GFP (pKW_R3). The cloned *gag* fragment is a sequence encoding the first 14 amino acids, including methionine. Thus, an additional start codon appears before the GFP. The Psi fragment is represented by the SL3 and SL4 loops of the HIV-1 genome. Then, site-directed adenine mutagenesis was conducted in the *gag* start codon in both plasmids (pKW_R1 and pKW_R3) to simulate base editing. As a result, two plasmids (pKW_R1m and pKW_R3m) were obtained, respectively. Schemes of the pKW and pKW_R series are presented in Supplementary Figure S6.

### 2.3. Plasmids Transfection

Cells (HEK293T or TZM-bl) were seeded in 12-well plates at a density of 180,000 per well in 1 mL of full medium (DMEM/F12, 10% FBS). After 12 h, cells were transfected with the necessary amount of DNA and the transfection reagent Lipofectamine 2000 (Thermo Fisher Scientific) in FBS-free Opti-MEM medium according to the manufacturer’s protocol. After 8 h, the medium was replaced with full fresh medium.

### 2.4. The Genome Editing Analysis by T7EI Assay

The genomic DNA of TZM-bl cells was extracted using a genomic DNA purification kit (#K0512, Thermo Fisher Scientific) according to the manufacturer’s protocol. Q5 High-Fidelity DNA Polymerase (New England Biolabs) and HIV-1-specific primers (F: 5′-TGTGACTCTGGTAACTAGAGATC-3′ and R: 5′-AGGCCAGGATTAACTGCGAAT-3′) were used to amplify the genomic DNA using PCR. PCR products were purified using a QIAquick gel extraction kit (QIAGEN, Hilden, Germany). The PCR products were detected by T7 Endonuclease I (T7EI) assay using an EnGen mutation detection kit (New England Biolabs) according to the manufacturer’s protocol.

### 2.5. Virus-like Particles Production

The production protocol described in the referenced research is used as the basis [15]. HEK293T cells were seeded in 10 cm dishes (TPP, Trasadingen, Switzerland) at a density of 3.2 × 10^6^ per plate in 10 mL of full medium. The next day, cells were transfected with Lipofectamine 2000 (Thermo Fisher Scientific) according to the manufacturer’s protocol. For production of VLPs with MmCas12m and dSpCas9, cells were transfected with a mixture of the plasmids VSV-G (400 ng, #8454 Addgene), psPAX2 (3375 ng, #12260 Addgene), pGag-MmCas12m-TadDE or pGag-dSpCas9-TadDE (1125 ng), and psgRNA-MmCas12m or psgRNA-dSpCas9 (4400 ng) in FBS-free Opti-MEM medium (Thermo Fisher Scientific). After 8 h, the medium was replaced with fresh production medium supplemented with 2% FBS (BioloT). The concentration was carried out according to the method described in the following reference [23]. Briefly, supernatants with VLPs (10 mL) were collected 48–50 h after transfection, centrifuged at 300× *g* for 3 min, filtered through a 0.45 μm PVDF filter (Millipore, Burlington, MA, USA), aliquoted in 2 mL tubes, and centrifuged at 21,000× *g* for 2.5 h (4 °C). A microcentrifuge with a fixed-angle rotor was used. Pellets with VLP samples were resuspended in Opti-MEM (Thermo Fisher Scientific), combined into one tube, and adjusted to 200 μL with Opti-MEM (approximate concentration by 50-fold). An aliquot (20 μL) of each sample was taken for Western blot, and the rest of the sample was treated with 1.8 μL of DNAse I (SibEnzyme, Novosibirsk, Russia) with the addition of the corresponding 10× DNAse I buffer by incubation at 37 °C for 10 min. VLP samples were stored at −70 °C.

### 2.6. Lentiviral Vector Production

The lentiviral vector was produced by the protocol described in the following reference [24]. Briefly, HEK293T cells at 3.2 × 10^6^ per plate (TPP) were placed in 10 mL of full medium. The cells were incubated at 37 °C and 5% CO_2_. The next day, cells were transfected with a packaging plasmid psPAX2 (#12260, Addgene), an envelope plasmid VSV-G (#8454, Addgene), and a transfer plasmid pLenti CMV GFP Puro (#17448, Addgene (1.64 pmol) using Lipofectamine 2000 (Thermo Fisher Scientific) in FBS-free Opti-MEM medium (Thermo Fisher Scientific) according to the manufacturer’s protocol. Total μg of the plasmid DNA was 27.8 μg in each sample. After 8 h, the medium was replaced with fresh production medium supplemented with 2% FBS (BioloT) and the cell culture was incubated at 37 °C and 5% CO_2_ for 48–50 h. Concentration was carried out in a similar manner to VLP samples. LVs served as a control for VLP electron microscopy.

### 2.7. VLPs Treatment

A total of 5 × 10^4^ TZM-bl cells in 50 µL were added to 50, 75, or 100 µL of VLPs, mixed by gentle pipetting, and cultured in a 96-well plate. After 8 h, the medium was replaced with fresh full medium. Cells were cultured. Genomic DNA was isolated on day 3 after VLP treatment.

### 2.8. NGS of TZM-bl DNA Samples

The following primers were used for the amplification of the edited locus of genome: F: 5′-AAAGGCGAGGGGCGGC-3′; R: 5′-TAACTGCGAATCGTTCTAGC-3′. The target locus was amplified using Phusion U Green Multiplex PCR Master Mix (Thermo Fisher Scientific) under the following conditions: 95 °C (3 min); 25 cycles of 95 °C (10 s), 57 °C (20 s) and 72 °C (20 s); and 72 °C (2 min). The PCR products were purified using a QIAquick gel extraction kit (QIAGEN). The NGS library preparation was carried out using a NEBNext Ultra II FS DNA library prep kit for Illumina (New England Biolabs). The sequencing was performed on the Illumina MiSeq platform (Illumina, San Diego, CA, USA). The NGS analysis was also carried out to search for the off-target effect using this protocol and specific primers.

### 2.9. Western Blot

To check the content of MmCas12m and dSpCas9 in HEK293T producer cells, cells were lysed 48 h after transfection in lysis buffer (45 mM Tris-HCl, pH 8.0, 160 mM NaCl, 5 mM EDTA, 1% (*w*/*v*) Triton X-100, and 1 mM phenylmethylsulfonyl fluoride). The lysis condition was 4 °C and 15 min. Lysate of each sample was centrifuged at 12,000× *g* at 4 °C for 10 min, supernatant was mixed with a 4× SDS-PAGE buffer (250 mM Tris-HCl, pH 6.8, 40% glycerol, 8% SDS, 4% 2-mercaptoethanol, and 0.2% Bromphenol Blue), and incubated at 95 °C for 3 min. To check the content of Cas in VLPs, concentrated samples were lysed in 4× SDS-PAGE buffer and incubated at 90 °C for 3 min.

SDS-PAGE was performed using 12% polyacrylamide gels in a Laemmli buffer system, followed by transfer onto a nitrocellulose membrane 0.45 µm (Bio-Rad, Hercules, CA, USA) using a power blotter system (#PB0012, Thermo Fisher Scientific). 6×His-tagged MmCas12m and dSpCas9 were detected using an anti-6×His tag mouse monoclonal antibody (#J099B12, BioLegend, San Diego, CA, USA); beta-actin (a loading control) was detected using a monoclonal mouse antibody (#GB15001, Dia-M, Moscow, Russia); and p24 HIV-1 (a loading control for VLPs samples) was detected using an anti-p24 mouse antibody (#MA5-44993, Thermo Fisher Scientific). A horseradish peroxidase-conjugated goat anti-mouse antibody (#1706516, Bio-Rad) was used as a secondary antibody. The image was obtained with the Gel Doc XR+ Gel Documentation System (Bio-Rad).

### 2.10. VLPs Electron Microscopy

The cell monolayer was fixed with a 4% paraformaldehyde solution and incubated at 4 °C for 24 h. The cell suspension was centrifuged in PBS 3 times at 500 rpm for 15 min. Then, the cells were fixed in OsO_4_ solution for 2 h and washed with PBS 3 times. Next, the cell cultures were dehydrated in an increasing concentration of ethanol battery (30%, 50%, 70%, 96%) for 15 min at each concentration. Then, the material was placed in a 1:1 mixture of 96% ethanol and acetone and incubated for 15 min, then incubated in 100% acetone for 15 min. The cells were placed in a mixture of acetone and epoxy resin (1:1) and incubated overnight. Then, the cells were fixed in epoxy resin (Embed + Araldite 502 + DDSA + DMP−0) (Electron Microscopy Sciences, Hatfield, PA, USA) and incubated for 48 h, 24 h at 48 °C and at 56 °C for 24 h. Ultrathin sections with a thickness of 40–60 nm were made using Reichert-Jung Ultracut E (Reichert Jung, Buffalo, NY, USA). The slices were placed on 200 mesh copper grids. The grids were placed in sections on a drop of solution of uranyl acetate and held for 15 min, then washed with distilled water and placed on a drop of lead citrate for 15 min. Visualization of concentrated VLPs and LVs was also performed. To do this, 10 μL of the sample was added to 200 mesh copper grids coated with a continuous carbon film. The sample was allowed to adsorb for 10 s. Excess solution was removed. Then, the grids were placed on a drop of solution of uranyl acetate and incubated for 20 s. The grids were dried and analyzed. VLPs/LVs were visualized using a JEM-1400 electron microscope (JEOL Ltd., Tokyo, Japan) at an accelerating voltage of 80 kV (Supplementary Figure S7).

### 2.11. Flow Cytometry

NovoCyte Quanteon 4025 flow cytometer systems (Agilent Technologies, Santa Clara, CA, USA) were used to analyze proportion of cells with fluorescence. The number of analyzed events in each sample is at least 5 × 10^4^.

### 2.12. Statistical Analysis

One-way ANOVA followed by Tukey’s post hoc test was used to compare differences between groups (*n* = 3 in each group). The level of statistical significance was set at *p* < 0.05. Statistical analyses were conducted using GraphPad Prism 9 software (v 9.3.1, San Diego, CA, USA).

### 2.13. DNA Plasmid Detection in Genomic DNA Samples

To identify the residual plasmid DNA used for VLP production in the genomic DNA samples, we performed RT-PCR screening using specific primers. Pairs of primers were chosen for the VSV-G gene (F: 5′-ATGAAGTGCCTTTTGTACTTAAC-3′, R: 5′-CTGTGCCTATTAAGTCATTATGCCA-3′), the MmCas12m (F: 5′-ACCATGACAGTGCATACAATG-3′, R: 5′-CTCGATTCTGGCCTGCTTCA-3′), and the dSpCas9 (F: 5′-TGATCACCGACGAGTACAAG-3′, R: 5′-AGGACTCTTCCAGTCTGTG-3′). RT-PCR was performed on a QuantStudio 5 thermocycler (Thermo Fisher Scientific) in 20 μL of BioMaster HS-Taq PCR reaction mix (Biolabmix, Novosibirsk, Russia) containing 0.4 pM of each primer and 0.2 pM of a probe under the following conditions: 95 °C (5 min); 45 cycles of 95 °C (10 seq), 57 °C (20 seq), and 72 °C (20 seq).

## 3. Results

### 3.1. Plasmids for Screening Cas Protein–DNA Interactions

At the first stage, screening plasmids were created to confirm the binding of the Cas-based ribonucleoprotein complex. pKW contains the *GFP* gene with a mutated Kozak sequence under the control of the CMV promoter. The mutated Kozak sequence provides a lower fluorescent signal. Based on pKW, we obtained pKW_R1 and pKW_R3 plasmids, composed of a fragment of the *gag* gene and the packaging signal (Psi) of HIV-1 before the *GFP* gene. Thus, the cloned HIV-1 fragment is located between the promoter and the GFP reporter. It is worth noting that the HIV-1 fragment is in a single reading frame with the GFP reporter in pKW_R1. And the same fragment is out of frame with GFP in pKW_R3.

**Figure 1:**
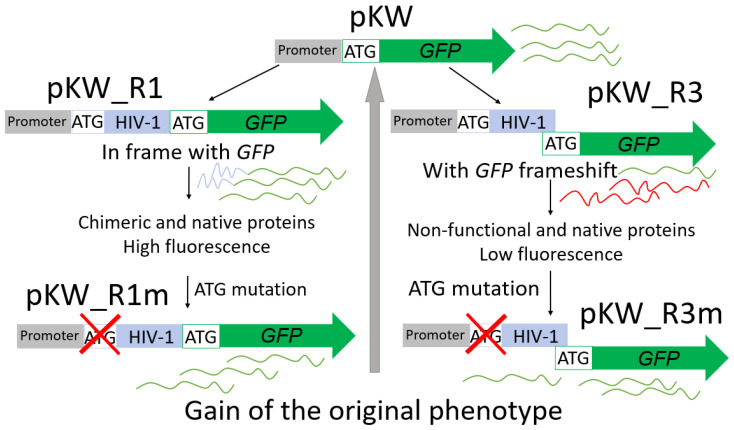
The scheme of the principle of the screening system. Detailed description in the text. HIV-1—fragment of the HIV-1 genome highlighted in blue; GFP—green fluorescent protein gene.

In case of successful targeting, the Cas-based complex will be on the path of RNA polymerase II movement. Therefore, fluorescence inhibition can be detected. Additionally, it is worth noting that the native start codon of the GFP reporter is preserved. Thus, the expression of the reporter gene is influenced by the state of the *gag* start codon. To confirm the operability of this system, mutagenesis (substitution G > A) was performed in the third position of the *gag* start codon while preserving the remaining nucleotide sequence of pKW_R1 and pKW_R3. Thus, pKW_R1m and pKW_R3m plasmids, respectively, were obtained (Figure 1).

**Figure 2:**
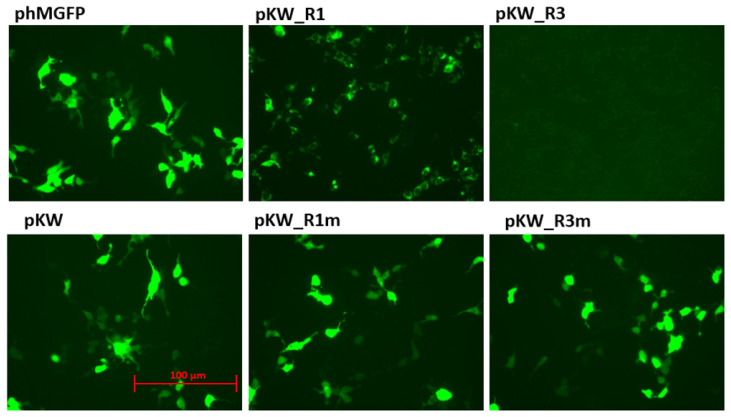
Characteristics of HEK293T cell fluorescence during transfection with phMGFP, pKW, pKW_R1, pKW_R1m, pKW_R3, and pKW_R3m plasmids.

The substitution G > A in the *gag* start codon simulates a mutation in the reverse chain (C > U during deamination) mediated by a base editor. To check the expression of the GFP reporter, HEK293T cells were transfected with all genetic constructs (200 ng each). Transfection results are shown in Figure 2.

**Figure 3:**
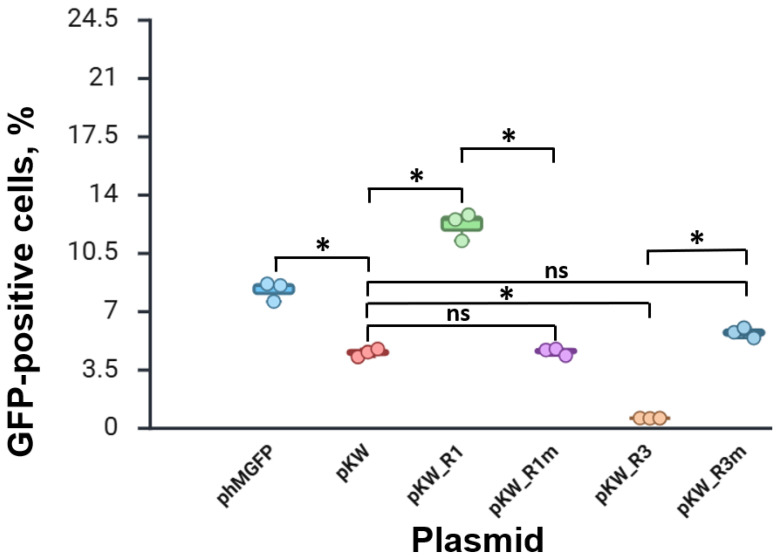
The results of HEK293T transfection with screening plasmids. Comparison of the GFP-positive cell proportions during expression for each plasmid of the screening system. * *p* < 0.05 based on Tukey’s test for all comparisons. ns—not significant.

It is noteworthy that the GFP was characterized by a specific fluorescence signal distribution during pKW_R1 transfection. Figure 2 shows that the fluorescent protein changed the localization pattern from the cytosol to the cytoplasmic membrane. This is due to the presence of a fragment of the HIV-1 gag protein consisting of a signal of the modification by myristic acid. This modification causes the nonspecific localization of the native full-size gag protein on the surface of the cell membrane [25]. Using flow cytometry, we quantified the proportion of cells with fluorescence in each experimental group (Figure 3, Supplementary Figure S8). pKW_R1 expression led to a threefold increase in the proportion of cells with fluorescence and pKW_R3 expression—on the contrary, the proportion of GFP-positive cells did not exceed 0.7%. Simulation of the disruption of the start codon (pKW_R1m and pKW_R3m plasmids) led to a rescue of the original phenotype characteristic of pKW. Thus, pKW_R1 and pKW_R3 constructs exhibit reporter expressions dependent on the state of the HIV-1 *gag* fragment.

### 3.2. In Vitro Testing of sgRNAs for Targeting MmCas12m to the Gag Start Codon Region

**Figure 4:**
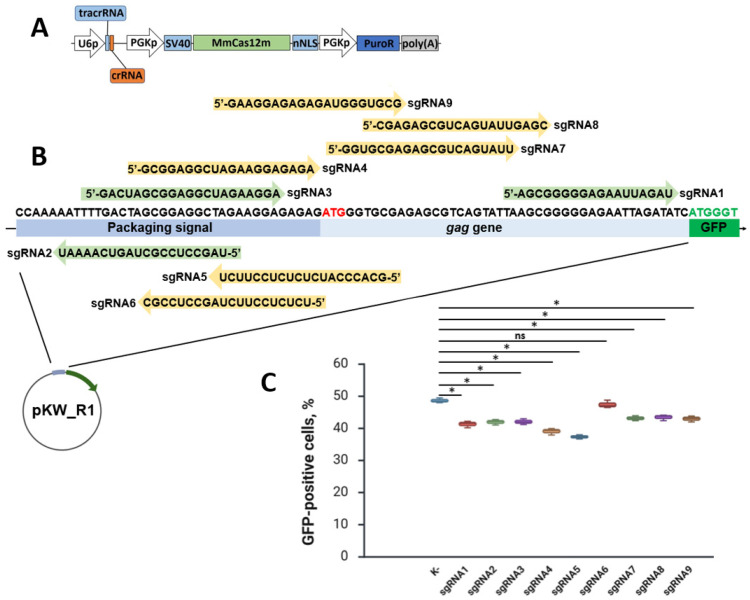
Application of the screening system to test MmCas12m sgRNAs to target HIV-1 DNA. (**A**) The image of the expression cassette of the pMmCas12m-sgRNA*N* plasmids used in the experiment; (**B**) the sequence fragment of the pKW_R1 plasmid containing HIV-1 DNA; (**C**) comparison of the GFP-positive cell proportions during pKW_R1 and pMmCas12m-sgRNA*N* co-transfection. Detailed description in the text. U6p—U6 promoter; MmCas12m—the *Mycolicibacterium mucogenicum* Cas12m gene; SV40—SV40 large T-antigen nuclear localization signal; nNLS—nucleoplasmin NLS; PuroR—the puromycin N-acetyl-transferase gene. (K-)—pKW_R1 and pMmCas12m-sgRNA without sgRNA co-transfected cells. sgRNAs with optimal PAMs highlighted in pale green. sgRNAs with non-canonical PAMs highlighted in pale yellow. *gag* start codon highlighted in red. * *p* < 0.05 based on Tukey’s test for all comparisons. ns—not significant.

Nine different sgRNAs were designed and cloned into pMmCas12m-sgRNAs. Obtained pMmCas12m-sgRNA*N* (N is the number of sgRNAs) plasmids code MmCas12m and one of nine sgRNAs. (Figure 4A). Notably, six of the nine sgRNAs have non-canonical PAM upstream from the target site (Figure 4B). In our case, we call a non-canonical PAM one which has a sequence other than TTN.

HEK293T cells were co-transfected with pMmCas12m-sgRNA and pKW_R1 (a molar ratio is 1:1) using Lipofectamine 2000. In theory, if the MmCas12m-sgRNA complex blocks transcription, the fluorescent signal will decrease. In this case, transcription inhibition is caused by the fact that the MmCas12m-sgRNA binds to DNA between the promoter and the *GFP* gene, thereby blocking the movement of RNA polymerase II. After 36 h of transfection, the number of GFP-positive cells was monitored using flow cytometry (Figure 4C). The use of sgRNA5 resulted in a decrease in the proportion of fluorescent HEK293T cells from about 49% (a control group—K-) to about 37%. The use of sgRNA1, 2, and 3 resulted in a decrease in the proportion of fluorescent HEK293T cells to about 41–42%, sgRNA4 to about 39.5%, and sgRNA7, 8, and 9 to about 43%. The use of sgRNA6 did not result in a statistically significant inhibition of transcription. At this stage, we have detected fluorescence inhibition, which may be caused by MmCas12m interference, at least for some sgRNAs. The same sgRNAs were also cloned into pdSpCas9-sgRNA, and co-transfection was also carried out using the same protocol. Fluorescence inhibition was detected for some dSpCas9 sgRNAs (Supplementary Figure S9). Thus, screening plasmids can be applied to various Cas proteins.

### 3.3. The TZM-bl Genome Editing

**Figure 5:**
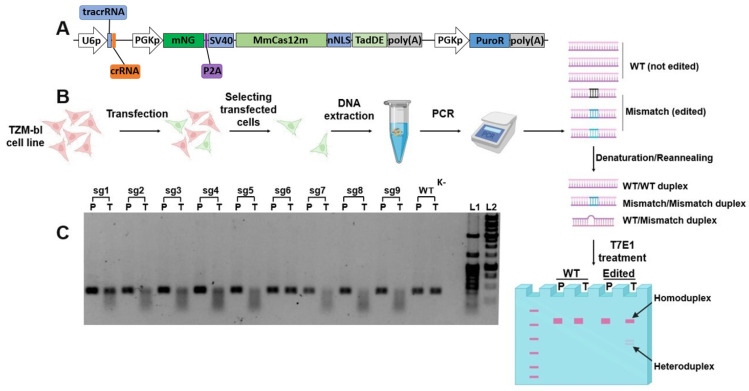
Genome editing analysis by T7EI assay. (**A**) Schematic of the expression cassete of the pMmCas12m-TadDE-sgRNA plasmid used in the experiment. U6p—U6 promoter; P2A—P2A self-cleaving peptide; MmCas12m—*Mycolicibacterium mucogenicum* Cas12m gene; SV40—SV40 large T-antigen nuclear localization signal; nNLS—nucleoplasmin NLS; TadDE—TadA-8e dual base editor gene; PGKp—phosphoglycerate kinase promoter; PuroR—puromycin N-acetyl-transferase gene. (**B**) Schematic of experiment stages. TZM-bl cell transfections with pMmCas12m-TadDE-sgRNA*N*, each encoding the MmCas12m-TadDE editing complex and one of the nine sgRNAs. Selection of transfected cells using puromycin. Extraction of the genomic DNA. Amplification of the edited locus using specific primers. Denaturation and reannealing of amplicons before T7EI treatment. In the presence of non-complementary duplexes, T7EI is active and introduces a double-stranded break. T7EI-treated amplicons are analyzed in agarose gel electrophoresis. (**C**) PCR (P) and T7EI assay (T) products of each edited group (1–9—sgRNA1-sgRNA9 respectively; WT—wild-type without editing). L1—100+ bp DNA marker; L2—1kb DNA marker. Detailed description in the text.

To pre-test the functionality of editing, sgRNAs were cloned into pMmCas12m-TadDE-sgRNA plasmids (Figure 5A). Each of the nine pMmCas12m-TadDE-sgRNA*N*s were transfected in pre-seeded TZM-bl cells. The TZM-bl cell line was previously obtained via the NIH AIDS reagent program. The TZM-bl cell line was obtained by lentivirus transduction and contains the 5′ region of the *gag* gene, including the start codon. sgRNAs were selected specifically to the start codon region of the *gag* gene. After 24 h of transfection, the medium was replaced with fresh medium containing puromycin to eliminate non-transfected cells (Figure 5B). After 36 h, genomic DNA was isolated. A PCR was conducted, covering a fragment of 372 bp surrounding the region of interest. Amplicons of each experimental sample were denatured and reannealed before the T7EI treatment (Figure 5B). If a mismatch occurs, the two strands of the DNA molecule will be cut into smaller fragments (Figure 5C). We detected edit events for all previously verified sgRNAs (numbers 1–9, excluding 6) within our screening plasmids.

**Figure 6:**
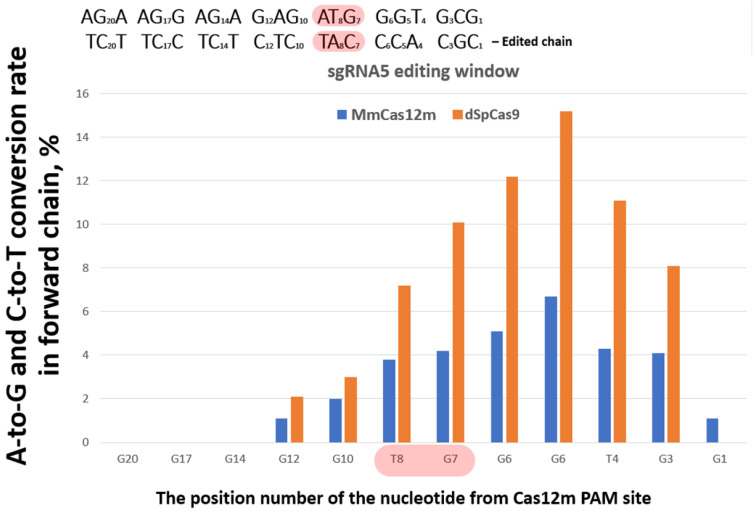
The editing efficiency of sgRNA5. Editing is mediated by plasmid transfection (blue is pMmCas12m-TadDE-sgRNA5, orange is pdSpCas9-TadDE-sgRNA5). Start codon nucleotides highlighted in red.

The isolated genomic DNA was used for the amplification of PCR products. The obtained amplicons were analyzed using the NGS method. Target mutations in the *gag* start codon were detected only when using sgRNA5 (Figure 6). Since sgRNA5 is complementary to the forward chain, the reverse chain changes into a single-stranded state when interacting with the editing complex. Consequently, the reverse chain was edited. The ATG start codon is complemented by the sequence 5′-CAT-3′ of the edited reverse chain. Mutations were detected in the position of nucleotide C in 4.25% of readings, and in the position of nucleotide A in 3.78%. The total efficiency of the disruption of the *gag* start codon was 8.03%. It is worth noting that the editing efficiency of plasmids coding MmCas12-TadDE for all sgRNA cases did not exceed 9%. To compare the editing efficiency, we used the well-annotated dSpCas9 in parallel (Supplementary Figure S10). For this purpose, similar sgRNAs were cloned into pdSpCas9-TadDE-sgRNA. Nine pdSpCas9-TadDE-sgRNA*N*s were transfected in the TZM-bl cells too. The data are presented for sgRNA5 due to its ability to disrupt the *gag* start codon (Figure 6). The effectiveness of editing when using other sgRNAs is shown in Supplementary Figure S10.

### 3.4. VLP-MmCas12m-TadDE Creation

**Figure 7:**
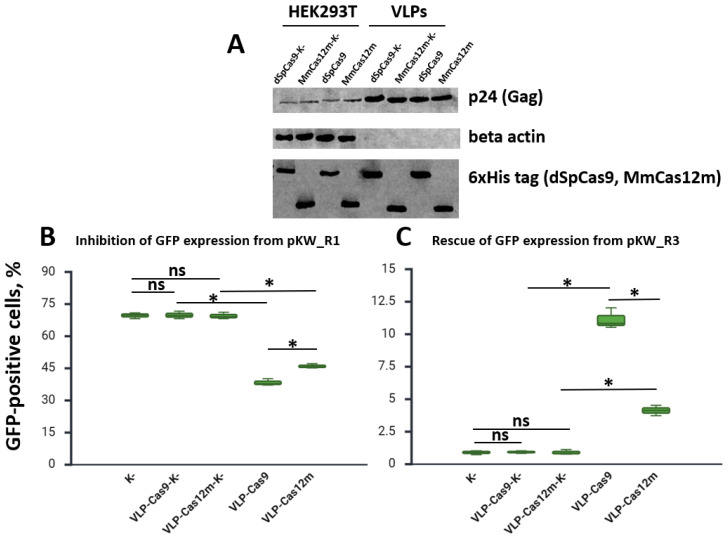
Generation of MmCas12m containing virus-like particles. (**A**) Representative Western blot evaluating the Cas content in lysates of HEK293T cells and purified VLPs. (**B**) Comparison of the GFP-positive cell proportions after transfection with pKW_R1 and VLPs delivery. (**C**) Comparison of the GFP-positive cell proportions after transfection with pKW_R3 and the VLP treatment. sgRNA5 is used for VLP production. (K-)—only transfected cells; (VLP-Cas9-K-, VLP-Cas12m-K-)—cells transfected and treated with VLP-dSpCas9-TadDE and VLP-MmCas12m-TadDE without sgRNA; (VLP-Cas9, VLP-Cas12m)—cells transfected and treated with VLP-dSpCas9-TadDE and VLP-MmCas12m-TadDE with sgRNA5. * *p* < 0.05 based on Tukey’s test for all comparisons. ns—not significant.

To produce VLP-MmCas12m-TadDE, we generated a plasmid (pGag-MmCas12m-TadDE) encoding 6×His-tagged MmCas12m-TadDE fused with the structural components of the lentivirus. We produced VLP-MmCas12m-TadDE in parallel with VLP-dSpCas9-TadDE. We used sgRNA5 for VLP production due to its ability to disrupt the *gag* start codon. VLPs were harvested and concentrated approximately 50-fold by centrifugation at 21,000× *g* for 2.5 h, using the protocol described in Section 2 and in the following reference [23]. We produced VLPs containing MmCas12m and dSpCas9 to target the *gag* start codon and analyzed Cas-based complex packaging into particles (Figure 7A). To pre-test the functionality of the VLPs, 8 h before delivery, HEK293T cells were transfected with pKW_R1 or pKW_R3 (Figure 7B,C and Figure S11).

On day 2 after VLP treatment, the number of GFP-positive cells with the 50 μL dose was measured by flow cytometry. The use of sgRNA5 resulted in a decrease in the proportion of fluorescent HEK293T cells (transfection with pKW_R1) from about 68% (control group) to about 47% with MmCas12m and about 37% with dSpCas9 (Supplementary Figure S12). We also detected an increase in the proportion of fluorescent HEK293T cells (transfection with pKW_R3) from about 1% (control group) to about 3.8% with MmCas12m and about 10–11% with dSpCas9 (Supplementary Figure S13).

### 3.5. VLP-Mediated Genome Editing

**Figure 8:**
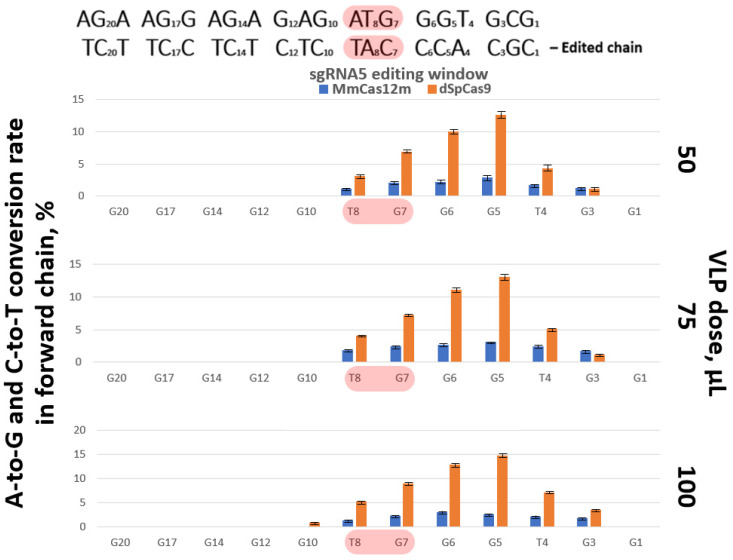
The editing efficiency of sgRNA5. Editing is mediated by the VLP treatment. Start codon nucleotides highlighted in red. Values and error bars represent the mean and standard deviation of three independent biological replicates.

The TZM-bl cells were treated with concentrated VLPs (50, 75 or 100 µL dose). After 72 h of incubation, the cells of each group were collected and genomic DNA was isolated. The isolated genomic DNA was used for the amplification of PCR products. The obtained amplicons were analyzed using the NGS method. Target mutations were detected in the position of nucleotide C in 2.21–2.91% of readings, and in the position of nucleotide A in 1.52–2.45%. The total efficiency of the disruption of the *gag* start is 3.73–5.36%, depending on the dose of VLPs (Figure 8, Supplementary Figure S14). It is worth noting that the editing efficiency of the start codon using VLP-MmCas12m-TadDE for all sgRNA cases does not exceed 8.9%. VLP-dSpCas9-TadDE-mediated editing provided better efficiency: 6.21–9.67% in the C position and 2.91–5.45% in the A position. The total efficiency of the disruption of the *gag* start codon is 9.12–15.12% in this case. The editing efficiency using VLP-dSpCas9-TadDE reached 24%, with some sgRNAs at some nucleotide positions.

We performed RT-PCR using specific primers to identify the residual plasmid DNA used for VLP production in samples of the genomic DNA. One pair of primers was selected for the VSV-G gene region and two pairs for the Cas encoding regions. However, no plasmid DNA was detected, which indicates the purity of the treated TZM-bl cells from the exogenous DNA.

We have predicted off-target sites using Cas-OFFinder software (v 2.4.1.) to identify DNA sequence similarity to the on-target site in the whole-human genome and the HIV-1 provirus. We suggested setting up mismatches for up to three nucleotides. Four potential off-target sites were identified for MmCas12m only in the human genome. Three off-target sites were identified for dSpCas9 only in the human genome (Supplementary Figure S15). We performed an NGS analysis of these loci after editing with VLP-MmCas12m-TadDE-sgRNA5 and VLP-dSpCas9-TadDE-sgRNA5. However, no de novo mutations have been identified. The search for off-target sites was conducted only for sgRNA5, as it ensured disruption of the *gag* start codon.

## 4. Discussion

Virus-like particles are a promising platform for delivering genomic editing tools. As part of this work, we have obtained virus-like particles based on lentivirus—VLP-MmCas12m-TadDE. The VLP-MmCas12m-TadDE is capable of delivering the MmCas12m-based ribonucleoprotein complex fused with a dual base editor into the mammalian cells in vitro.

Plasmids of the pKW_R series demonstrated effectiveness for testing Cas protein–DNA interactions. According to our results, the use of pKW_R plasmids is effective for different DNA-specific Cas proteins. We successfully applied pKW_R1 to identify the effect of MmCas12m and dSpCas9 on GFP-reporter expression. We detected a different inhibitory ability depending on the Cas protein used. The advantage of using pKW_R plasmids is the simplicity and speed of obtaining preliminary results for sgRNA selection or confirmation of Cas protein–DNA interaction. It is worth noting that pKW_R1 is better suited for detecting inhibition mediated by CRISPR interference. pKW_R3 is better suited for revealing the functionality of the base editor. We do not exclude the use of screening plasmids to test prime editors, for example. The researcher can clone any sequence before GFP, depending on the task. Similar screening plasmids have been used in other studies and have proven themselves well [21,26,27,28]. The general principle is an easily detectable change in fluorescence after editing.

Of all the tested sgRNAs, only sgRNA5 provided disruption of the *gag* start codon. During plasmid transfection-mediated editing, the efficiency was about 9% in the case of MmCas12m-TadDE. It is worth noting that, adenine base editor fused with another Cas12m variant (GoCas12m under the control of the CAG promoter) provided editing efficiency of about 10% in HEK293 cells in another original work [21]. However, the editing efficiency reached 38% if we used plasmids encoding dSpCas9-TadDE. dSpCas9-TadDE provided the greater editing efficiency during transfection-mediated editing (Supplementary Figure S9). This level of editing is acceptable for the SpCas9 variant in the case of non-canonical PAM (different from NGG) [26]. SaCas9 is another widely used variant. The SaCas9-mediated base editor efficiency varies from about 20 to 58% [29,30]. SauriCas9 was also used for base editing. The maximum editing efficiency was about 40% [31]. Cas12a (Cpf1) is another Cas12 protein that requires a T-rich PAM sequence (TTTV) for target-DNA recognition. For this reason, Cas12a was originally used to enhance the editing capability [32]. However, Cas12a still has a large size (1307 amino acids) comparable to SpCas9 (1368 amino acids). Efficiency of Cas12a-mediated base editors can reach 40% [27]. The efficiency of Cas12a-mediated editing can reach about 70% with the modification of deaminases [33]. We used TadDE deaminase, which was obtained during the original study [22]. In the original study, the editing efficiency was 73–80% under the optimal conditions. However, they used the nickase form of SpCas9. Indeed, the nickase forms of Cas proteins are widely used to improve the editing efficiency. However, the use of nickase forms may be associated with genotoxicity [34]. Consequently, using dCas and naturally inactive MmCas12m is a deliberate safety-first approach to eliminate indels. Accordingly, we applied MmCas12m for base editing to induce accurate substitutions without indels. In this regard, dSpCas9 was used for correct comparison with MmCas12m. The use of nSpCas9-TadDE in our other experiments ensured editing at the level of 60–70% under the condition of canonical PAM. According to the experimental data presented here, under the condition of optimal PAM, dSpCas9 provided an efficiency 2–3 times higher than MmCas12m in transfection-mediated editing. At the same time, both editors had a similar sized editing window.

A similar pattern of efficiency was observed in VLP-mediated editing. The use of VLP-MmCas12m-TadDE provided lower efficiency than VLP-dSpCas9-TadDE. Notably, the higher dose had no effect on the efficiency of VLP-MmCas12m-TadDE-mediated editing. On the contrary, the higher dose of VLP-dSpCas9-TadDE led not only to an increase in the efficiency, but also to an expansion of the editing window (Figure 8). However, it is worth noting a few details. MmCas12m provided inhibition of the GFP reporter on a par with dSpCas9, and in some cases even better. In addition, MmCas12m proved to be the variant less susceptible to deviations from optimal PAM than dSpCas9 (Figure 4, Supplementary Figure S9). The fact that dSpCas9 responded to the dose but MmCas12m did not suggests that VLP packaging or ribonucleoprotein stability, rather than just concentration, might be the limiting factor for MmCas12m. According to the Western blot analysis, MmCas12m-TadDE is successfully packaged in VLPs. Perhaps a low level of effectiveness is a specific feature of MmCas12m proteins. It can be assumed that the specific structure of MmCas12m blocks the exposure of the NLS for efficient transport into the nucleus. It is possible that the variation of the NLSs and their locations in the structure of the editing complex may change the editing efficiency. Additionally, a special linker may be required for MmCas12m. It makes sense to test different linkers: the XTEN linker, long and short glycine–serine linkers, and rigid proline linkers. A future research direction will focus on explaining this.

Nevertheless, our results show the prospect of using VLPs for the delivery of ribonucleoprotein complexes. Further work will be aimed at improving the efficiency by modifying the components of the ribonucleoprotein editing complex and optimizing VLP production. Despite the low editing efficiency, we experimentally confirmed the production and functionality of the original VLPs designed to deliver MmCas12m-based ribonucleoprotein complexes into mammalian cells in vitro. The created VLP-MmCas12m-TadDE has a morphology similar to lentivirus according to the results of electron microscopy. This indicates a common structure (Supplementary Figure S7). VLP-MmCas12m-TadDE successfully incorporates the full-size editing complex and can be used for in vitro treatment. The results obtained will not only form the basis for subsequent work, but will also be useful to all those working towards HIV-1 gene therapy.

## 5. Conclusions

This is the first time we have used VLPs to deliver a new MmCas12m-TadDE dual base editor to the *gag* start codon. We have disrupted the *gag* start codon using our VLP-MmCas12m-TadDE. These results can serve as a basis for further research in the field of HIV-1 gene therapy. Unfortunately, the editing efficiency is low compared to VLP-dSpCas9-TadDE. At the moment, few studies have been conducted with Cas12m proteins for base editing. In this regard, it is difficult to give an unambiguous answer. However, an assumption can be made based on the available data. Perhaps Cas12m, a miniature type V CRISPR effector, exhibits low base editing efficiency due to its inherent nature as a high-affinity DNA-binding protein rather than an active nuclease, limiting catalytic turnover. It is possible that the use of special linkers and NLSs will increase efficiency. A future research direction will focus on explaining this.

## Figures and Tables

**Table 1:** List of plasmids used.

Plasmid Name	Description
psPAX2 (#12260, Addgene)	Lentiviral packaging plasmid
pCMV-VSV-G (#8454, Addgene)	Lentiviral envelope plasmid
pLenti CMV GFP Puro (#17448, Addgene)	Lentiviral transfer plasmid
dSpCas9 (#201953, Addgene)	Mammalian expression plasmid of catalytically inactive SpCas9
SpCas9_sgRNA (#122089, Addgene)	U6 driven sgRNA expression vector for cloning own guides
phMGFP	Codes green fluorescent protein under the control of the CMV promoter
pKW	Identical to phMGFP, but Kozak sequence has been replaced from GCCACCAUG to GATATCAUG
pKW_R1	Identical to pKW, but contains a *gag* gene fragment of the HIV genome in-frame with GFP
pKW_R1m	Identical to pKW_R1, but contains a mutation in the start codon of the *gag* gene fragment
pKW_R3	Identical to pKW, but contains a *gag* gene fragment of the HIV genome with reading frameshift of GFP
pKW_R3m	Identical to pKW_R3, but contains a mutation in the start codon of the *gag* gene
pMmCas12m-sgRNA*N*	Codes sgRNA and MmCas12m, *N* is number of sgRNAs
pdSpCas9-sgRNA*N*	Codes sgRNA and catalytically inactive SpCas9, *N* is number of sgRNAs
pMmCas12m-TadDE-sgRNA*N*	Codes sgRNA, MmCas12m and TadA-8e dual base editor (TadDE), *N* is number of sgRNAs
pdSpCas9-TadDE-sgRNA*N*	Codes sgRNA, catalytically inactive SpCas9 and TadA-8e dual base editor (TadDE), *N* is number of sgRNAs
pGag-MmCas12m-TadDE	Codes VLP-Cas12m-TadDE components fused with 6×His tag
pGag-dSpCas9-TadDE	Codes VLP-dSpCas9-TadDE components fused with 6×His tag
psgRNA-MmCas12m	U6-driven MmCas12m sgRNA expression vector for cloning own guides for VLP production
psgRNA-dSpCas9	U6-driven SpCas9 sgRNA expression vector for cloning own guides for VLP production

## Data Availability

Eukaryotic cell lines were obtained from the cell culture collection of the Federal Budgetary Institution of Science, “State Research Center of Virology and Biotechnology ‘Vector’”, Russia. The original contributions presented in this study are included in the article and Supplementary Materials. Further inquiries can be directed to the corresponding author.

## Supplementary Materials

The following supporting information can be downloaded at: https://www.mdpi.com/article/10.3390/cimb48030241/s1, Figure S1: The DNA sequence of the MmCas12m-TadDE editing complex; Figure S2: The DNA sequence of the dSpCas9-TadDE editing complex; Figure S3: The DNA sequence of the Gag-MmCas12m-TadDE fusion protein; Figure S4: The DNA sequence of the Gag-dSpCas9-TadDE fusion protein; Figure S5: DNA fragments used to create pMmCas12m-TadDE-sgRNA, psgRNA-MmCas12m, pdSpCas9-TadDE-sgRNA, and psgRNA-dSpCas9; Figure S6: Maps for custom plasmids pKW and pKW_R; Figure S7: Electron microscopic images of purified virus-like particles and lentiviruses, including VLP budding; Figure S8: The results of HEK293T transfection with screening plasmids: phMGFP, pKW, pKW_R1, pKW_R3, pKW_R1m, and pKW_R3m; Figure S9: Application of the screening system to test dSpCas9 sgRNAs to target HIV-1 DNA; Figure S10: The editing efficiency with sgRNA 1–4, 6–9. Editing is mediated by plasmid transfection; Figure S11: The scheme of inhibition of transcription using VLPs; Figure S12: Comparison of the GFP-positive cell proportions after transfection with pKW_R1 and the VLP treatment; Figure S13: Comparison of the GFP-positive cell proportions after transfection with pKW_R3 and the VLP treatment; Figure S14: The editing efficiency of start codon nucleotides with sgRNA5. Editing is mediated by VLP treatment; Figure S15: The list of potential off-target sites during sgRNA5-mediated editing.

## Abbreviations

The following abbreviations are used in this manuscript:

VLPs	Virus-like particles
sgRNA	Single-guide RNA
AAV	Adeno-associated virus
HIV	Human immunodeficiency virus
VSV	Vesicular stomatitis virus
CMV	Cytomegalovirus
TadDE	TadA-8e dual base editor
LV	Lentivirus
FBS	Fetal bovine serum
GFP	Green fluorescent protein
NLS	Nuclear localization signal
NGS	Next-generation sequencing
PCR	Polymerase chain reaction
PBS	Phosphate-buffered saline
Tris	Tris(hydroxymethyl)aminomethane
SDS-PAGE	Sodium dodecyl sulfate–polyacrylamide gel electrophoresis
PAM	Protospacer adjacent motif
