# Supplementary material for: The Disruption of the HIV-1 Gag Start Codon via Editing Using MmCas12m-Dual Base Editor-Loaded Virus-like Particles
Source: Curr Issues Mol Biol. 2026 Feb 25;48(3):241. doi: 10.3390/cimb48030241 (PMC13025988; doi:10.3390/cimb48030241)
Supplement: Supplementary file 1 [file cimb-48-00241-s001.zip › cimb-4153435-supplementary.pdf]

GGGGCCCAAGAAAAAGCGGAAAGTGGGGCCCAGCGGCACCACCATGACAGTGCATACAATGGGCGTGCAC
TACAAGTGGCAGATCCCCGAGGTTCTGAGACAGCAGCTGTGGCTGGCCCACAACCTGAGAGAAGATCTGGT
GTCTCTGCAGCTGGCCTACGACGACGACCTGAAGGCCATCTGGTCTAGCTACCCTGATGTGGCCCAGGCCGA
GGATACAATGGCTGCCGCTGAAGCTGATGCCGTGGCTCTGTCTGAGAGAGTGAAGCAGGCCAGAATCGAGG
CCAGAAGCAAGAAGATCAGCACCGAGCTGACCCAGCAGCTGAGGGACGCCAAGAAGAGACTGAAGGACG
CCAGACAGGCCAGACGGGATGCCATTGCCGTGGTCAAAGATGATGCCGCCGAGCGGAGAAAGGCCAGATC
TGATCAACTGGCCGCCGACCAGAAAGCCCTGTACGGCCAGTACTGCAGAGATGGCGATCTGTACTGGGCCA
GCTTCAACACCGTGCTGGACCACCACAAGACCGCCGTGAAGAGAATCGCCGCTCAGAGAGCCTCTGGCAAG
CCTGCCACACTGAGACACCACAGATTTGACGGCAGCGGCACAATCGCCGTGCAACTCCAAAGACAAGCTGG
CGCCCCTCCTCGGACACCTATGGTTCTTGCTGATGAGGCCGGCAAGTACCGGAACGTGCTGCACATTCCTGG
CTGGACCGATCCTGATGTGTGGGAGCAGATGACACGGTCCCAGTGCAGACAGTCTGGCAGAGTGACAGTGC
GGATGAGATGCGGCAGCACAGATGGACAGCCCCAGTGGATCGATCTGCCTGTGCAGGTTCACAGATGGCTG
CCTGCCGACGCCGATATTACAGGCGCTGAGCTGGTGGTCACAAGAGTGGCCGGAATCTACAGAGCCAAGCT
GTGCGTGACCGCCAGGATCGGAGATACCGAGCCTGTGACATCTGGCCCTACAGTGGCCCTGCATCTTGGCTG
GCGATCTACCGAAGAGGGAACAGCCGTGGCCACTTGGAGATCTGATGCCCCTCTGGACATCCCCTTCGGCCT
GAGAACAGTGATGAGAGTGGATGCTGCCGGCACCTCTGGCATCATTGTGGTGCCTGCCACCATCGAGAGAA
GGCTGACCCGGACAGAGAATATCGCCAGCAGCAGATCTCTGGCCCTGGACGCCCTGAGAGATAAGGTTGTC
GGCTGGCTGAGCGACAACGACGCCCCTACCTATAGAGATGCTCCACTGGAAGCCGCCACCGTGAAGCAGTG
GAAGTCCCCTCAGAGATTCGCCTCTCTGGCTCACGCCTGGAAGGACAATGGCACCGAGATCAGCGATATCCT
GTGGGCCTGGTTCAGCCTGGACAGAAAGCAGTGGGCTCAGCAAGAGAACGGCAGAAGAAAGGCCCTGGGA
CACAGAGATGACCTGTATCGGCAGATCGCCGCCGTGATCTCTGATCAGGCTGGACACGTGCTGGTGGACGAT
ACAAGTGTGGCCGAGCTGAGCGCCAGAGCTATGGAAAGAACCGAGCTGCCTACCGAGGTGCAGCAGAAGA
TCGACAGAAGAAGGACCACGCTGCTCCCGGTGGACTGAGAGCTTCTGTGGTGGCCGCCATGACAAGAGAT
GGGGTGCCAGTGACAATCGTGGCCGCAGCCGATTTCACCCGGACACACAGCAGATGCGGACACGTGAACCC
CGCCGACGATAGATACCTGAGCAACCCCGTCAGATGCGACGGCTGTGGCGCTATGTACGACCAGGACAGAT
CCTTCGTGACCCTGATGCTGAGAGCCGCCACAGCTCCTAGCAATCCTGGCAAAAGACCTGCCGCCACAAAG
AAGGCCGGACAGGCCAAAAAGAAAAAGGGTCTCAGCGGTGGTAGCAGCGGCGGGCCTACCAGCGAAGTGG
AATTCAGCCATGAATATTGGATGCGTCATGCACTGACCCTGGCAAAACGTGCCCGTGATGAAGGTGAAGCA
CCGGTTGGTGCAGTTCTGGTTCTGAATAATCGTGTTATTGGTGAAGGTTGGAATCGTCGTATTGGTCTGCATG
ATCCGACCGCACATGCAGAAATTATGGCACTGCGTCAAGGTGGTCTGGTTATGCAGAATAGCCGTCTGATTG
ATGCAACCCTGTATGTTACCTTTGAACCGTGTGTTATGTGTGCCGGTGCAATGATTAATAGCCGCATTGGTCG
TGTTGTTTTTGGTGTTCGTAATAGCAAACGTGGTGCAGCAGGTAGCCTGATGAATGTTCTGAATTATCCGGGT
ATGAATCACCGTGTTGAAATTACCGAAGGTATTCTGGCAGATGAATGTGCAGCACTGCTGTGTGATTTTTATC
GTATGCCTCGTCAGGTTTTTAACGCACAGAAAAAGGCACAGAGCAGCATTAAT

Supplementary Figure S1. The complete nucleotide sequence coding for the MmCas12m-TadDE editing complex. Red = SV40 large T-antigen nuclear localization signal (NLS); Blue = the *Mycolicibacterium mucogenicum* Cas12m; Orange = nucleoplasmin NLS; Violet = the glycine-serine linker; Green = the TadA-8e dual base editor.

GGGGCCCAAGAAAAAGCGGAAAGTGGACAAGAAGTACAGCATCGGCCTGGCCATCGGCACCAACTCTGTG
GGCTGGGCCGTGATCACCGACGAGTACAAGGTGCCCAGCAAGAAATTCAAGGTGCTGGGCAACACCGACC
GGCACAGCATCAAGAAGAACCTGATCGGAGCCCTGCTGTTCGACAGCGGCGAAACAGCCGAGGCCACCCG
GCTGAAGAGAACCGCCAGAAGAAGATACACCAGACGGAAGAACCGGATCTGCTATCTGCAAGAGATCTTC
AGCAACGAGATGGCCAAGGTGGACGACAGCTTCTTCCACAGACTGGAAGAGTCCTTCCTGGTGGAAGAGGA
TAAGAAGCACGAGCGGCACCCCATCTTCGGCAACATCGTGGACGAGGTGGCCTACCACGAGAAGTACCCCA
CCATCTACCACCTGAGAAAGAAACTGGTGGACAGCACCGACAAGGCCGACCTGCGGCTGATCTATCTGGCC
CTGGCCCACATGATCAAGTTCCGGGGCCACTTCCTGATCGAGGGCGACCTGAACCCCGACAACAGCGACGT
GGACAAGCTGTTCATCCAGCTGGTGCAGACCTACAACCAGCTGTTCGAGGAAAACCCCATCAACGCCAGCG
GCGTGGACGCCAAGGCCATCCTGTCTGCCAGACTGAGCAAGAGCAGACGGCTGGAAAATCTGATCGCCCAG
CTGCCCGGCGAGAAGAAGAATGGCCTGTTCGGAAACCTGATTGCCCTGAGCCTGGGCCTGACCCCCAACTTC
AAGAGCAACTTCGACCTGGCCGAGGATGCCAAACTGCAGCTGAGCAAGGACACCTACGACGACGACCTGG
ACAACCTGCTGGCCCAGATCGGCGACCAGTACGCCGACCTGTTTCTGGCCGCCAAGAACCTGTCCGACGCC
ATCCTGCTGAGCGACATCCTGAGAGTGAACACCGAGATCACCAAGGCCCCCCTGAGCGCCTCTATGATCAA
GAGATACGACGAGCACCACCAGGACCTGACCCTGCTGAAAGCTCTCGTGCGGCAGCAGCTGCCTGAGAAGT
ACAAAGAGATTTTCTTCGACCAGAGCAAGAACGGCTACGCCGGCTACATTGACGGCGGAGCCAGCCAGGA
AGAGTTCTACAAGTTCATCAAGCCCATCCTGGAAAAGATGGACGGCACCGAGGAACTGCTCGTGAAGCTGA
ACAGAGAGGACCTGCTGCGGAAGCAGCGGACCTTCGACAACGGCAGCATCCCCCACCAGATCCACCTGGG
AGAGCTGCACGCCATTCTGCGGCGGCAGGAAGATTTTTACCCATTCCTGAAGGACAACCGGGAAAAGATCG
AGAAGATCCTGACCTTCCGCATCCCCTACTACGTGGGCCCTCTGGCCAGGGGAAACAGCAGATTCGCCTGGA
TGACCAGAAAGAGCGAGGAAACCATCACCCCCTGGAACTTCGAGGAAGTGGTGGACAAGGGCGCTTCCGC
CCAGAGCTTCATCGAGCGGATGACCAACTTCGATAAGAACCTGCCCAACGAGAAGGTGCTGCCCAAGCACA
GCCTGCTGTACGAGTACTTCACCGTGTATAACGAGCTGACCAAAGTGAAATACGTGACCGAGGGAATGAGA
AAGCCCGCCTTCCTGAGCGGCGAGCAGAAAAAGGCCATCGTGGACCTGCTGTTCAAGACCAACCGGAAAGT
GACCGTGAAGCAGCTGAAAGAGGACTACTTCAAGAAAATCGAGTGCTTCGACTCCGTGGAAATCTCCGGCG
TGGAAGATCGGTTCAACGCCTCCCTGGGCACATACCACGATCTGCTGAAAATTATCAAGGACAAGGACTTCC
TGGACAATGAGGAAAACGAGGACATTCTGGAAGATATCGTGCTGACCCTGACACTGTTTGAGGACAGAGAG
ATGATCGAGGAACGGCTGAAAACCTATGCCCACCTGTTCGACGACAAAGTGATGAAGCAGCTGAAGCGGCG
GAGATACACCGGCTGGGGCAGGCTGAGCCGGAAGCTGATCAACGGCATCCGGGACAAGCAGTCCGGCAAG
ACAATCCTGGATTTCCTGAAGTCCGACGGCTTCGCCAACAGAAACTTCATGCAGCTGATCCACGACGACAGC
CTGACCTTTAAAGAGGACATCCAGAAAGCCCAGGTGTCCGGCCAGGGCGATAGCCTGCACGAGCACATTGC
CAATCTGGCCGGCAGCCCCGCCATTAAGAAGGGCATCCTGCAGACAGTGAAGGTGGTGGACGAGCTCGTGA
AAGTGATGGGCCGGCACAAGCCCGAGAACATCGTGATCGAAATGGCCAGAGAGAACCAGACCACCCAGAA
GGGACAGAAGAACAGCCGCGAGAGAATGAAGCGGATCGAAGAGGGCATCAAAGAGCTGGGCAGCCAGAT
CCTGAAAGAACACCCCGTGGAAAACACCCAGCTGCAGAACGAGAAGCTGTACCTGTACTACCTGCAGAATG
GGCGGGATATGTACGTGGACCAGGAACTGGACATCAACCGGCTGTCCGACTACGATGTGGACGCCATCGTG
CCTCAGAGCTTTCTGAAGGACGACTCCATCGACAACAAGGTGCTGACCAGAAGCGACAAGAACCGGGGCA
AGAGCGACAACGTGCCCTCCGAAGAGGTCGTGAAGAAGATGAAGAACTACTGGCGGCAGCTGCTGAACGC
CAAGCTGATTACCCAGAGAAAGTTCGACAATCTGACCAAGGCCGAGAGAGGCGGCCTGAGCGAACTGGAT
AAGGCCGGCTTCATCAAGAGACAGCTGGTGGAAACCCGGCAGATCACAAAGCACGTGGCACAGATCCTGG
ACTCCCGGATGAACACTAAGTACGACGAGAATGACAAGCTGATCCGGGAAGTGAAAGTGATCACCCTGAA
GTCCAAGCTGGTGTCCGATTTCCGGAAGGATTTCCAGTTTTACAAAGTGCGCGAGATCAACAACTACCACCA
CGCCCACGACGCCTACCTGAACGCCGTCGTGGGAACCGCCCTGATCAAAAAGTACCCTAAGCTGGAAAGCG
AGTTCGTGTACGGCGACTACAAGGTGTACGACGTGCGGAAGATGATCGCCAAGAGCGAGCAGGAAATCGG
CAAGGCTACCGCCAAGTACTTCTTCTACAGCAACATCATGAACTTTTTCAAGACCGAGATTACCCTGGCCAA
CGGCGAGATCCGGAAGCGGCCTCTGATCGAGACAAACGGCGAAACCGGGGAGATCGTGTGGGATAAGGGC
CGGGATTTTGCCACCGTGCGGAAAGTGCTGAGCATGCCCCAAGTGAATATCGTGAAAAAGACCGAGGTGCA
GACAGGCGGCTTCAGCAAAGAGTCTATCCTGCCCAAGAGGAACAGCGATAAGCTGATCGCCAGAAAGAAG
GACTGGGACCCTAAGAAGTACGGCGGCTTCGACAGCCCCACCGTGGCCTATTCTGTGCTGGTGGTGGCCAAA
GTGGAAAAGGGCAAGTCCAAGAAACTGAAGAGTGTGAAAGAGCTGCTGGGGATCACCATCATGGAAAGAA
GCAGCTTCGAGAAGAATCCCATCGACTTTCTGGAAGCCAAGGGCTACAAAGAAGTGAAAAAGGACCTGATC
ATCAAGCTGCCTGAGTACTCCCTGTTCGAGCTGGAAAACGGCCGGAAGAGAATGCTGGCCTCTGCCGGCGA

ACTGCAGAAGGGAAACGAACTGGCCCTGCCCTCCAAATATGTGAACTTCCTGTACCTGGCCAGCCACTATGA
GAAGCTGAAGGGCTCCCCCGAGGATAATGAGCAGAAACAGCTGTTTGTGGAACAGCACAAGCACTACCTGG
ACGAGATCATCGAGCAGATCAGCGAGTTCTCCAAGAGAGTGATCCTGGCCGACGCTAATCTGGACAAAGTG
CTGTCCGCCTACAACAAGCACCGGGATAAGCCCATCAGAGAGCAGGCCGAGAATATCATCCACCTGTTTAC
CCTGACCAATCTGGGAGCCCCTGCCGCCTTCAAGTACTTTGACACCACCATCGACCGGAAGAGGTACACCA
GCACCAAAGAGGTGCTGGACGCCACCCTGATCCACCAGAGCATCACCGGCCTGTACGAGACACGGATCGAC
CTGTCTCAGCTGGGAGGCGACAAAAGACCTGCCGCCACAAAGAAGGCCGGACAGGCCAAAAAGAAAAAG
GGTCTCAGCGGTGGTAGCAGCGGCGGGCCTACCAGCGAAGTGGAATTCAGCCATGAATATTGGATGCGTCA
TGCACTGACCCTGGCAAAACGTGCCCGTGATGAAGGTGAAGCACCGGTTGGTGCAGTTCTGGTTCTGAATAA
TCGTGTTATTGGTGAAGGTTGGAATCGTCGTATTGGTCTGCATGATCCGACCGCACATGCAGAAATTATGGCA
CTGCGTCAAGGTGGTCTGGTTATGCAGAATAGCCGTCTGATTGATGCAACCCTGTATGTTACCTTTGAACCGT
GTGTTATGTGTGCCGGTGCAATGATTAATAGCCGCATTGGTCGTGTTGTTTTTGGTGTTCGTAATAGCAAACGT
GGTGCAGCAGGTAGCCTGATGAATGTTCTGAATTATCCGGGTATGAATCACCGTGTTGAAATTACCGAAGGT
ATTCTGGCAGATGAATGTGCAGCACTGCTGTGTGATTTTTATCGTATGCCTCGTCAGGTTTTTAACGCACAGA
AAAAGGCACAGAGCAGCATTAAT

Supplementary Figure S2. The complete nucleotide sequence coding for the dSpCas9-TadDE editing complex. Red = SV40 large T-antigen nuclear localization signal (NLS); Blue = the catalytically inactive *Streptococcus pyogenes* Cas9; Orange = nucleoplasmin NLS; Violet = glycine-serine linker; Green = the TadA-8e dual base editor.

ATGGGTGCGAGAGCGTCAGTATTAAGCGGGGGAGAATTAGATCGATGGGAAAAAATTCGGTTAAGGCCAGG
GGGAAAGAAAAAATATAAATTAAAACATATAGTATGGGCAAGCAGGGAGCTAGAACGATTCGCAGTTAAT
CCTGGCCTGTTAGAAACATCAGAAGGCTGTAGACAAATACTGGGACAGCTACAACCATCCCTTCAGACAGG
ATCAGAAGAACTTAGATCATTATATAATACAGTAGCAACCCTCTATTGTGTGCATCAAAGGATAGAGATAAA
AGACACCAAGGAAGCTTTAGACAAGATAGAGGAAGAGCAAAACAAAAGTAAGAAAAAAGCACAGCAAGC
AGCAGCTGACACAGGACACAGCAATCAGGTCAGCCAAAATTACCCTATAGTGCAGAACATCCAGGGGCAA
ATGGTACATCAGGCCATATCACCTAGAACTTTAAATGCATGGGTAAAAGTAGTAGAAGAGAAGGCTTTCAG
CCCAGAAGTGATACCCATGTTTTCAGCATTATCAGAAGGAGCCACCCCACAAGATTTAAACACCATGCTAA
ACACAGTGGGGGGACATCAAGCAGCCATGCAAATGTTAAAAGAGACCATCAATGAGGAAGCTGCAGAATG
GGATAGAGTGCATCCAGTGCATGCAGGGCCTATTGCACCAGGCCAGATGAGAGAACCAAGGGGAAGTGAC
ATAGCAGGAACTACTAGTACCCTTCAGGAACAAATAGGATGGATGACACATAATCCACCTATCCCAGTAGG
AGAAATCTATAAAAGATGGATAATCCTGGGATTAAATAAAATAGTAAGAATGTATAGCCCTACCAGCATTC
TGGACATAAGACAAGGACCAAAGGAACCCTTTAGAGACTATGTAGACCGATTCTATAAAACTCTAAGAGCC
GAGCAAGCTTCACAAGAGGTAAAAAATTGGATGACAGAAACCTTGTTGGTCCAAAATGCGAACCCAGATTG
TAAGACTATTTTAAAAGCATTGGGACCAGGAGCGACACTAGAAGAAATGATGACAGCATGTCAGGGAGTGG
GGGGACCCGGCCATAAAGCAAGAGTTTTGGCTGAAGCAATGAGCCAAGTAACAAATCCAGCTACCATAATG
ATACAGAAAGGCAATTTTAGGAACCAAAGAAAGACTGTTAAGTGTTTCAATTGTGGCAAAGAAGGGCACAT
AGCCAAAAATTGCAGGGCCCCTAGGAAAAAGGGCTGTTGGAAATGTGGAAAGGAAGGACACCAAATGAAA
GATTGTACTGAGAGACAGGCTAATTTTTTAGGGAAGATCTGGCCTTCCCACAAGGGAAGGCCAGGGAATTTT
CTTCAGAGCAGACCAGAGCCAACAGCCCCACCAGAAGAGAGCTTCAGGTTTGGGGAAGAGACAACAACTC
CCTCTCAGAAGCAGGAGCCGATAGACAAGGAACTGTATCCTTTAGCTTCCCTCAGATCACTCTTTGGCAGCG
ACCCCTCGTCACAATAAAGATAGGGGGGCAATTAAAGGAAGCTCTATTAGATACAGGAGCAGATGATACAG
TATTAGAAGAAATGAATTTGCCAGGAAGATGGAAACCAAAAATGATAGGGGGAATTGGAGGTTTTATCAAA
GTAAGACAGTATGATCAGATACTCATAGAAATCTGCGGACATAAAGCTATAGGTACAGTATTAGTAGGACC
TACACCTGTCAACATAATTGGAAGAAATCTGTTGACTCAGATTGGCTGCACTTTAAATTTTCCCATTAGTCCT
GGGGGGCCCAAGAAAAAGCGGAAAGTGGGGCCCAGCGGCACCACCATGACAGTGCATACAATGGGCGTGC
ACTACAAGTGGCAGATCCCCGAGGTTCTGAGACAGCAGCTGTGGCTGGCCCACAACCTGAGAGAAGATCTG
GTGTCTCTGCAGCTGGCCTACGACGACGACCTGAAGGCCATCTGGTCTAGCTACCCTGATGTGGCCCAGGCC
GAGGATACAATGGCTGCCGCTGAAGCTGATGCCGTGGCTCTGTCTGAGAGAGTGAAGCAGGCCAGAATCGA
GGCCAGAAGCAAGAAGATCAGCACCGAGCTGACCCAGCAGCTGAGGGACGCCAAGAAGAGACTGAAGGA
CGCCAGACAGGCCAGACGGGATGCCATTGCCGTGGTCAAAGATGATGCCGCCGAGCGGAGAAAGGCCAGA
TCTGATCAACTGGCCGCCGACCAGAAAGCCCTGTACGGCCAGTACTGCAGAGATGGCGATCTGTACTGGGC
CAGCTTCAACACCGTGCTGGACCACCACAAGACCGCCGTGAAGAGAATCGCCGCTCAGAGAGCCTCTGGCA
AGCCTGCCACACTGAGACACCACAGATTTGACGGCAGCGGCACAATCGCCGTGCAACTCCAAAGACAAGCT
GGCGCCCCTCCTCGGACACCTATGGTTCTTGCTGATGAGGCCGGCAAGTACCGGAACGTGCTGCACATTCCT
GGCTGGACCGATCCTGATGTGTGGGAGCAGATGACACGGTCCCAGTGCAGACAGTCTGGCAGAGTGACAGT
GCGGATGAGATGCGGCAGCACAGATGGACAGCCCCAGTGGATCGATCTGCCTGTGCAGGTTCACAGATGGC
TGCCTGCCGACGCCGATATTACAGGCGCTGAGCTGGTGGTCACAAGAGTGGCCGGAATCTACAGAGCCAAG
CTGTGCGTGACCGCCAGGATCGGAGATACCGAGCCTGTGACATCTGGCCCTACAGTGGCCCTGCATCTTGGC
TGGCGATCTACCGAAGAGGGAACAGCCGTGGCCACTTGGAGATCTGATGCCCCTCTGGACATCCCCTTCGGC
CTGAGAACAGTGATGAGAGTGGATGCTGCCGGCACCTCTGGCATCATTGTGGTGCCTGCCACCATCGAGAGA
AGGCTGACCCGGACAGAGAATATCGCCAGCAGCAGATCTCTGGCCCTGGACGCCCTGAGAGATAAGGTTGT
CGGCTGGCTGAGCGACAACGACGCCCCTACCTATAGAGATGCTCCACTGGAAGCCGCCACCGTGAAGCAGT
GGAAGTCCCCTCAGAGATTCGCCTCTCTGGCTCACGCCTGGAAGGACAATGGCACCGAGATCAGCGATATC
CTGTGGGCCTGGTTCAGCCTGGACAGAAAGCAGTGGGCTCAGCAAGAGAACGGCAGAAGAAAGGCCCTGG
GACACAGAGATGACCTGTATCGGCAGATCGCCGCCGTGATCTCTGATCAGGCTGGACACGTGCTGGTGGAC
GATACAAGTGTGGCCGAGCTGAGCGCCAGAGCTATGGAAAGAACCGAGCTGCCTACCGAGGTGCAGCAGA
AGATCGACAGAAGAAGGGACCACGCTGCTCCCGGTGGACTGAGAGCTTCTGTGGTGGCCGCCATGACAAGA
GATGGGGTGCCAGTGACAATCGTGGCCGCAGCCGATTTCACCCGGACACACAGCAGATGCGGACACGTGAA
CCCCGCCGACGATAGATACCTGAGCAACCCCGTCAGATGCGACGGCTGTGGCGCTATGTACGACCAGGACA
GATCCTTCGTGACCCTGATGCTGAGAGCCGCCACAGCTCCTAGCAATCCTGGCAAAAGACCTGCCGCCACA
AAGAAGGCCGGACAGGCCAAAAAGAAAAAGGGTCTCAGCGGTGGTAGCAGCGGCGGGCCTACCAGCGAA

GTGGAATTCAGCCATGAATATTGGATGCGTCATGCACTGACCCTGGCAAAACGTGCCCGTGATGAAGGTGA
AGCACCGGTTGGTGCAGTTCTGGTTCTGAATAATCGTGTTATTGGTGAAGGTTGGAATCGTCGTATTGGTCTG
CATGATCCGACCGCACATGCAGAAATTATGGCACTGCGTCAAGGTGGTCTGGTTATGCAGAATAGCCGTCTG
ATTGATGCAACCCTGTATGTTACCTTTGAACCGTGTGTTATGTGTGCCGGTGCAATGATTAATAGCCGCATTG
GTCGTGTTGTTTTTGGTGTTCGTAATAGCAAACGTGGTGCAGCAGGTAGCCTGATGAATGTTCTGAATTATCC
GGGTATGAATCACCGTGTTGAAATTACCGAAGGTATTCTGGCAGATGAATGTGCAGCACTGCTGTGTGATTTT
TATCGTATGCCTCGTCAGGTTTTTAACGCACAGAAAAAGGCACAGAGCAGCATTAATCATCACCATCACCAT
CAC

Supplementary Figure S3. The complete nucleotide sequence coding for the Gag-MmCas12m-TadDE fusion protein. Black = HIV-1 Gag; Red = SV40 large T-antigen nuclear localization signal; Blue = the *Mycolicibacterium mucogenicum* Cas12m; Orange = nucleoplasmin NLS; Violet = the glycine-serine linker; Green = the TadA-8e dual base editor, Red = 6xHis tag.

ATGGGTGCGAGAGCGTCAGTATTAAGCGGGGGAGAATTAGATCGATGGGAAAAAATTCGGTTAAGGCCAGG
GGGAAAGAAAAAATATAAATTAAAACATATAGTATGGGCAAGCAGGGAGCTAGAACGATTCGCAGTTAAT
CCTGGCCTGTTAGAAACATCAGAAGGCTGTAGACAAATACTGGGACAGCTACAACCATCCCTTCAGACAGG
ATCAGAAGAACTTAGATCATTATATAATACAGTAGCAACCCTCTATTGTGTGCATCAAAGGATAGAGATAAA
AGACACCAAGGAAGCTTTAGACAAGATAGAGGAAGAGCAAAACAAAAGTAAGAAAAAAGCACAGCAAGC
AGCAGCTGACACAGGACACAGCAATCAGGTCAGCCAAAATTACCCTATAGTGCAGAACATCCAGGGGCAA
ATGGTACATCAGGCCATATCACCTAGAACTTTAAATGCATGGGTAAAAGTAGTAGAAGAGAAGGCTTTCAG
CCCAGAAGTGATACCCATGTTTTCAGCATTATCAGAAGGAGCCACCCCACAAGATTTAAACACCATGCTAA
ACACAGTGGGGGGACATCAAGCAGCCATGCAAATGTTAAAAGAGACCATCAATGAGGAAGCTGCAGAATG
GGATAGAGTGCATCCAGTGCATGCAGGGCCTATTGCACCAGGCCAGATGAGAGAACCAAGGGGAAGTGAC
ATAGCAGGAACTACTAGTACCCTTCAGGAACAAATAGGATGGATGACACATAATCCACCTATCCCAGTAGG
AGAAATCTATAAAAGATGGATAATCCTGGGATTAAATAAAATAGTAAGAATGTATAGCCCTACCAGCATTC
TGGACATAAGACAAGGACCAAAGGAACCCTTTAGAGACTATGTAGACCGATTCTATAAAACTCTAAGAGCC
GAGCAAGCTTCACAAGAGGTAAAAAATTGGATGACAGAAACCTTGTTGGTCCAAAATGCGAACCCAGATTG
TAAGACTATTTTAAAAGCATTGGGACCAGGAGCGACACTAGAAGAAATGATGACAGCATGTCAGGGAGTGG
GGGGACCCGGCCATAAAGCAAGAGTTTTGGCTGAAGCAATGAGCCAAGTAACAAATCCAGCTACCATAATG
ATACAGAAAGGCAATTTTAGGAACCAAAGAAAGACTGTTAAGTGTTTCAATTGTGGCAAAGAAGGGCACAT
AGCCAAAAATTGCAGGGCCCCTAGGAAAAAGGGCTGTTGGAAATGTGGAAAGGAAGGACACCAAATGAAA
GATTGTACTGAGAGACAGGCTAATTTTTTAGGGAAGATCTGGCCTTCCCACAAGGGAAGGCCAGGGAATTTT
CTTCAGAGCAGACCAGAGCCAACAGCCCCACCAGAAGAGAGCTTCAGGTTTGGGGAAGAGACAACAACTC
CCTCTCAGAAGCAGGAGCCGATAGACAAGGAACTGTATCCTTTAGCTTCCCTCAGATCACTCTTTGGCAGCG
ACCCCTCGTCACAATAAAGATAGGGGGGCAATTAAAGGAAGCTCTATTAGATACAGGAGCAGATGATACAG
TATTAGAAGAAATGAATTTGCCAGGAAGATGGAAACCAAAAATGATAGGGGGAATTGGAGGTTTTATCAAA
GTAAGACAGTATGATCAGATACTCATAGAAATCTGCGGACATAAAGCTATAGGTACAGTATTAGTAGGACC
TACACCTGTCAACATAATTGGAAGAAATCTGTTGACTCAGATTGGCTGCACTTTAAATTTTCCCATTAGTCCT
GGGGGGCCCAAGAAAAAGCGGAAAGTGGACAAGAAGTACAGCATCGGCCTGGCCATCGGCACCAACTCTG
TGGGCTGGGCCGTGATCACCGACGAGTACAAGGTGCCCAGCAAGAAATTCAAGGTGCTGGGCAACACCGAC
CGGCACAGCATCAAGAAGAACCTGATCGGAGCCCTGCTGTTCGACAGCGGCGAAACAGCCGAGGCCACCC
GGCTGAAGAGAACCGCCAGAAGAAGATACACCAGACGGAAGAACCGGATCTGCTATCTGCAAGAGATCTT
CAGCAACGAGATGGCCAAGGTGGACGACAGCTTCTTCCACAGACTGGAAGAGTCCTTCCTGGTGGAAGAGG
ATAAGAAGCACGAGCGGCACCCCATCTTCGGCAACATCGTGGACGAGGTGGCCTACCACGAGAAGTACCCC
ACCATCTACCACCTGAGAAAGAAACTGGTGGACAGCACCGACAAGGCCGACCTGCGGCTGATCTATCTGGC
CCTGGCCCACATGATCAAGTTCCGGGGCCACTTCCTGATCGAGGGCGACCTGAACCCCGACAACAGCGACG
TGGACAAGCTGTTCATCCAGCTGGTGCAGACCTACAACCAGCTGTTCGAGGAAAACCCCATCAACGCCAGC
GGCGTGGACGCCAAGGCCATCCTGTCTGCCAGACTGAGCAAGAGCAGACGGCTGGAAAATCTGATCGCCCA
GCTGCCCGGCGAGAAGAAGAATGGCCTGTTCGGAAACCTGATTGCCCTGAGCCTGGGCCTGACCCCCAACTT
CAAGAGCAACTTCGACCTGGCCGAGGATGCCAAACTGCAGCTGAGCAAGGACACCTACGACGACGACCTG
GACAACCTGCTGGCCCAGATCGGCGACCAGTACGCCGACCTGTTTCTGGCCGCCAAGAACCTGTCCGACGC
CATCCTGCTGAGCGACATCCTGAGAGTGAACACCGAGATCACCAAGGCCCCCCTGAGCGCCTCTATGATCA
AGAGATACGACGAGCACCACCAGGACCTGACCCTGCTGAAAGCTCTCGTGCGGCAGCAGCTGCCTGAGAAG
TACAAAGAGATTTTCTTCGACCAGAGCAAGAACGGCTACGCCGGCTACATTGACGGCGGAGCCAGCCAGGA
AGAGTTCTACAAGTTCATCAAGCCCATCCTGGAAAAGATGGACGGCACCGAGGAACTGCTCGTGAAGCTGA
ACAGAGAGGACCTGCTGCGGAAGCAGCGGACCTTCGACAACGGCAGCATCCCCCACCAGATCCACCTGGG
AGAGCTGCACGCCATTCTGCGGCGGCAGGAAGATTTTTACCCATTCCTGAAGGACAACCGGGAAAAGATCG
AGAAGATCCTGACCTTCCGCATCCCCTACTACGTGGGCCCTCTGGCCAGGGGAAACAGCAGATTCGCCTGGA
TGACCAGAAAGAGCGAGGAAACCATCACCCCCTGGAACTTCGAGGAAGTGGTGGACAAGGGCGCTTCCGC
CCAGAGCTTCATCGAGCGGATGACCAACTTCGATAAGAACCTGCCCAACGAGAAGGTGCTGCCCAAGCACA
GCCTGCTGTACGAGTACTTCACCGTGTATAACGAGCTGACCAAAGTGAAATACGTGACCGAGGGAATGAGA
AAGCCCGCCTTCCTGAGCGGCGAGCAGAAAAAGGCCATCGTGGACCTGCTGTTCAAGACCAACCGGAAAGT
GACCGTGAAGCAGCTGAAAGAGGACTACTTCAAGAAAATCGAGTGCTTCGACTCCGTGGAAATCTCCGGCG

TGGAAGATCGGTTCAACGCCTCCCTGGGCACATACCACGATCTGCTGAAAATTATCAAGGACAAGGACTTCC
TGGACAATGAGGAAAACGAGGACATTCTGGAAGATATCGTGCTGACCCTGACACTGTTTGAGGACAGAGAG
ATGATCGAGGAACGGCTGAAAACCTATGCCCACCTGTTCGACGACAAAGTGATGAAGCAGCTGAAGCGGCG
GAGATACACCGGCTGGGGCAGGCTGAGCCGGAAGCTGATCAACGGCATCCGGGACAAGCAGTCCGGCAAG
ACAATCCTGGATTTCCTGAAGTCCGACGGCTTCGCCAACAGAAACTTCATGCAGCTGATCCACGACGACAGC
CTGACCTTTAAAGAGGACATCCAGAAAGCCCAGGTGTCCGGCCAGGGCGATAGCCTGCACGAGCACATTGC
CAATCTGGCCGGCAGCCCCGCCATTAAGAAGGGCATCCTGCAGACAGTGAAGGTGGTGGACGAGCTCGTGA
AAGTGATGGGCCGGCACAAGCCCGAGAACATCGTGATCGAAATGGCCAGAGAGAACCAGACCACCCAGAA
GGGACAGAAGAACAGCCGCGAGAGAATGAAGCGGATCGAAGAGGGCATCAAAGAGCTGGGCAGCCAGAT
CCTGAAAGAACACCCCGTGGAAAACACCCAGCTGCAGAACGAGAAGCTGTACCTGTACTACCTGCAGAATG
GGCGGGATATGTACGTGGACCAGGAACTGGACATCAACCGGCTGTCCGACTACGATGTGGACGCCATCGTG
CCTCAGAGCTTTCTGAAGGACGACTCCATCGACAACAAGGTGCTGACCAGAAGCGACAAGAACCGGGGCA
AGAGCGACAACGTGCCCTCCGAAGAGGTCGTGAAGAAGATGAAGAACTACTGGCGGCAGCTGCTGAACGC
CAAGCTGATTACCCAGAGAAAGTTCGACAATCTGACCAAGGCCGAGAGAGGCGGCCTGAGCGAACTGGAT
AAGGCCGGCTTCATCAAGAGACAGCTGGTGGAAACCCGGCAGATCACAAAGCACGTGGCACAGATCCTGG
ACTCCCGGATGAACACTAAGTACGACGAGAATGACAAGCTGATCCGGGAAGTGAAAGTGATCACCCTGAA
GTCCAAGCTGGTGTCCGATTTCCGGAAGGATTTCCAGTTTTACAAAGTGCGCGAGATCAACAACTACCACCA
CGCCCACGACGCCTACCTGAACGCCGTCGTGGGAACCGCCCTGATCAAAAAGTACCCTAAGCTGGAAAGCG
AGTTCGTGTACGGCGACTACAAGGTGTACGACGTGCGGAAGATGATCGCCAAGAGCGAGCAGGAAATCGG
CAAGGCTACCGCCAAGTACTTCTTCTACAGCAACATCATGAACTTTTTCAAGACCGAGATTACCCTGGCCAA
CGGCGAGATCCGGAAGCGGCCTCTGATCGAGACAAACGGCGAAACCGGGGAGATCGTGTGGGATAAGGGC
CGGGATTTTGCCACCGTGCGGAAAGTGCTGAGCATGCCCCAAGTGAATATCGTGAAAAAGACCGAGGTGCA
GACAGGCGGCTTCAGCAAAGAGTCTATCCTGCCCAAGAGGAACAGCGATAAGCTGATCGCCAGAAAGAAG
GACTGGGACCCTAAGAAGTACGGCGGCTTCGACAGCCCCACCGTGGCCTATTCTGTGCTGGTGGTGGCCAAA
GTGGAAAAGGGCAAGTCCAAGAAACTGAAGAGTGTGAAAGAGCTGCTGGGGATCACCATCATGGAAAGAA
GCAGCTTCGAGAAGAATCCCATCGACTTTCTGGAAGCCAAGGGCTACAAAGAAGTGAAAAAGGACCTGATC
ATCAAGCTGCCTGAGTACTCCCTGTTCGAGCTGGAAAACGGCCGGAAGAGAATGCTGGCCTCTGCCGGCGA
ACTGCAGAAGGGAAACGAACTGGCCCTGCCCTCCAAATATGTGAACTTCCTGTACCTGGCCAGCCACTATGA
GAAGCTGAAGGGCTCCCCCGAGGATAATGAGCAGAAACAGCTGTTTGTGGAACAGCACAAGCACTACCTGG
ACGAGATCATCGAGCAGATCAGCGAGTTCTCCAAGAGAGTGATCCTGGCCGACGCTAATCTGGACAAAGTG
CTGTCCGCCTACAACAAGCACCGGGATAAGCCCATCAGAGAGCAGGCCGAGAATATCATCCACCTGTTTAC
CCTGACCAATCTGGGAGCCCCTGCCGCCTTCAAGTACTTTGACACCACCATCGACCGGAAGAGGTACACCA
GCACCAAAGAGGTGCTGGACGCCACCCTGATCCACCAGAGCATCACCGGCCTGTACGAGACACGGATCGAC
CTGTCTCAGCTGGGAGGCGACAAAAGACCTGCCGCCACAAAGAAGGCCGGACAGGCCAAAAAGAAAAAG
GGTCTCAGCGGTGGTAGCAGCGGCGGGCCTACCAGCGAAGTGGAATTCAGCCATGAATATTGGATGCGTCA
TGCACTGACCCTGGCAAAACGTGCCCGTGATGAAGGTGAAGCACCGGTTGGTGCAGTTCTGGTTCTGAATAA
TCGTGTTATTGGTGAAGGTTGGAATCGTCGTATTGGTCTGCATGATCCGACCGCACATGCAGAAATTATGGCA
CTGCGTCAAGGTGGTCTGGTTATGCAGAATAGCCGTCTGATTGATGCAACCCTGTATGTTACCTTTGAACCGT
GTGTTATGTGTGCCGGTGCAATGATTAATAGCCGCATTGGTCGTGTTGTTTTTGGTGTTCGTAATAGCAAACGT
GGTGCAGCAGGTAGCCTGATGAATGTTCTGAATTATCCGGGTATGAATCACCGTGTTGAAATTACCGAAGGT
ATTCTGGCAGATGAATGTGCAGCACTGCTGTGTGATTTTTATCGTATGCCTCGTCAGGTTTTTAACGCACAGA
AAAAGGCACAGAGCAGCATTAATCATCACCATCACCATCAC

Supplementary Figure S4. The complete nucleotide sequence coding for the Gag-dSpCas9-TadDE fusion protein. Black = HIV-1 Gag; Red = SV40 large T-antigen nuclear localization signal; Blue = the catalytically inactive *Streptococcus pyogenes* Cas9; Orange = nucleoplasmin NLS; Violet = the glycine-serine linker; Green = the TadA-8e dual base editor, Red = 6xHis tag.

A

GAGGGCCTATTTCCCATGATTCCTTCATATTTGCATATACGATACAAGGCTGTTAGAGAGATAATTGGAATTA
ATTTGACTGTAAACACAAAGATATTAGTACAAAATACGTGACGTAGAAAGTAATAATTTCTTGGGTAGTTTG
CAGTTTTAAAATTATGTTTTAAAATGGACTATCATATGCTTACCGTAACTTGAAAGTATTTCGATTTCTTGGCT
TTATATATCTTGTGGAAAGGACGAAACACCGTGTCATAGCCCAGCTTGGCGGGCGAAGGCCAAGACGGAGA
CCGACACAAGCTTCAGTGGTCTCTTTTTTTTT

B

GAGGGCCTATTTCCCATGATTCCTTCATATTTGCATATACGATACAAGGCTGTTAGAGAGATAATTGGAATTA
ATTTGACTGTAAACACAAAGATATTAGTACAAAATACGTGACGTAGAAAGTAATAATTTCTTGGGTAGTTTG
CAGTTTTAAAATTATGTTTTAAAATGGACTATCATATGCTTACCGTAACTTGAAAGTATTTCGATTTCTTGGCT
TTATATATCTTGTGGAAAGGACGAAACACCGGAGACCGACACAAGCTTCAGTGGTCTCTGTTTAAGAGCTAT
GCTGGAAACAGCATAGCAAGTTTAAATAAGGCTAGTCCGTTATCAACTTGAAAAAGTGGCACCGAGTCGGT
GC

Supplementary Figure S5: DNA fragments used to create pMmCas12m-TadDE-sgRNA, psgRNA-MmCas12m (A), and pdSpCas9-TadDE-sgRNA, psgRNA-dSpCas9 (B). Black = the U6 promoter, Violet = RNA scaffold for MmCas12m, Blue = RNA scaffold for dSpCas9, Red = the fragment with BsaI cloning sites.

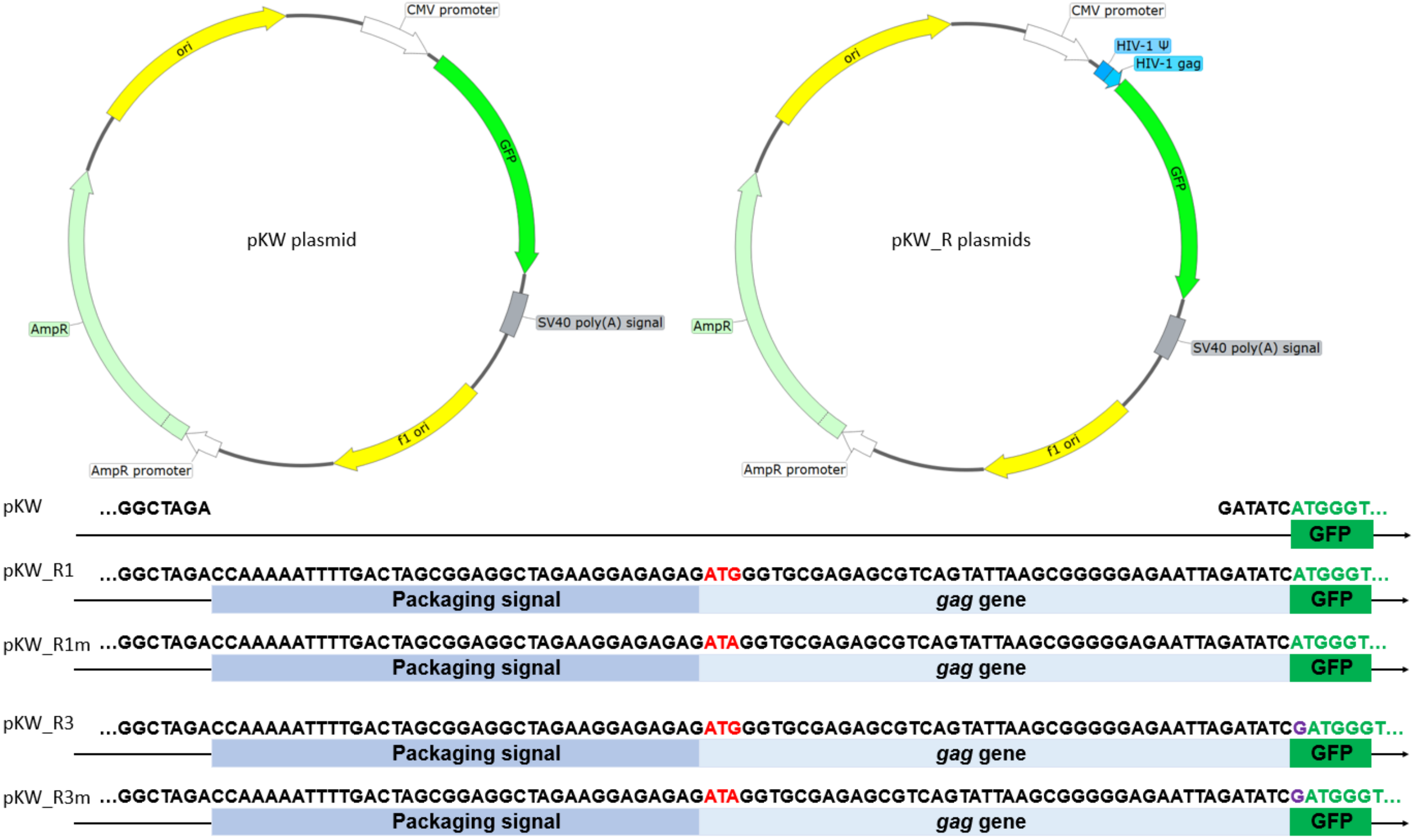

Supplementary Figure S6: Maps for custom plasmids pKW and pKW_R. The Psi and *gag* sequences cloned in plasmids are presented. *gag* start codon highlighted in red. The violet G nucleotide leads to a frameshift mutation. GFP – green fluorescent protein gene, SV40 poly(A) signal – SV40 polyadenylation signal, f1 ori – f1 bacteriophage origin of replication, AmpR promoter is responsible for initiating the transcription of the ampicillin resistance gene, AmpR – beta-lactamase gene, ori – high-copy-number ColE1/pMB1/pBR322/pUC origin of replication.

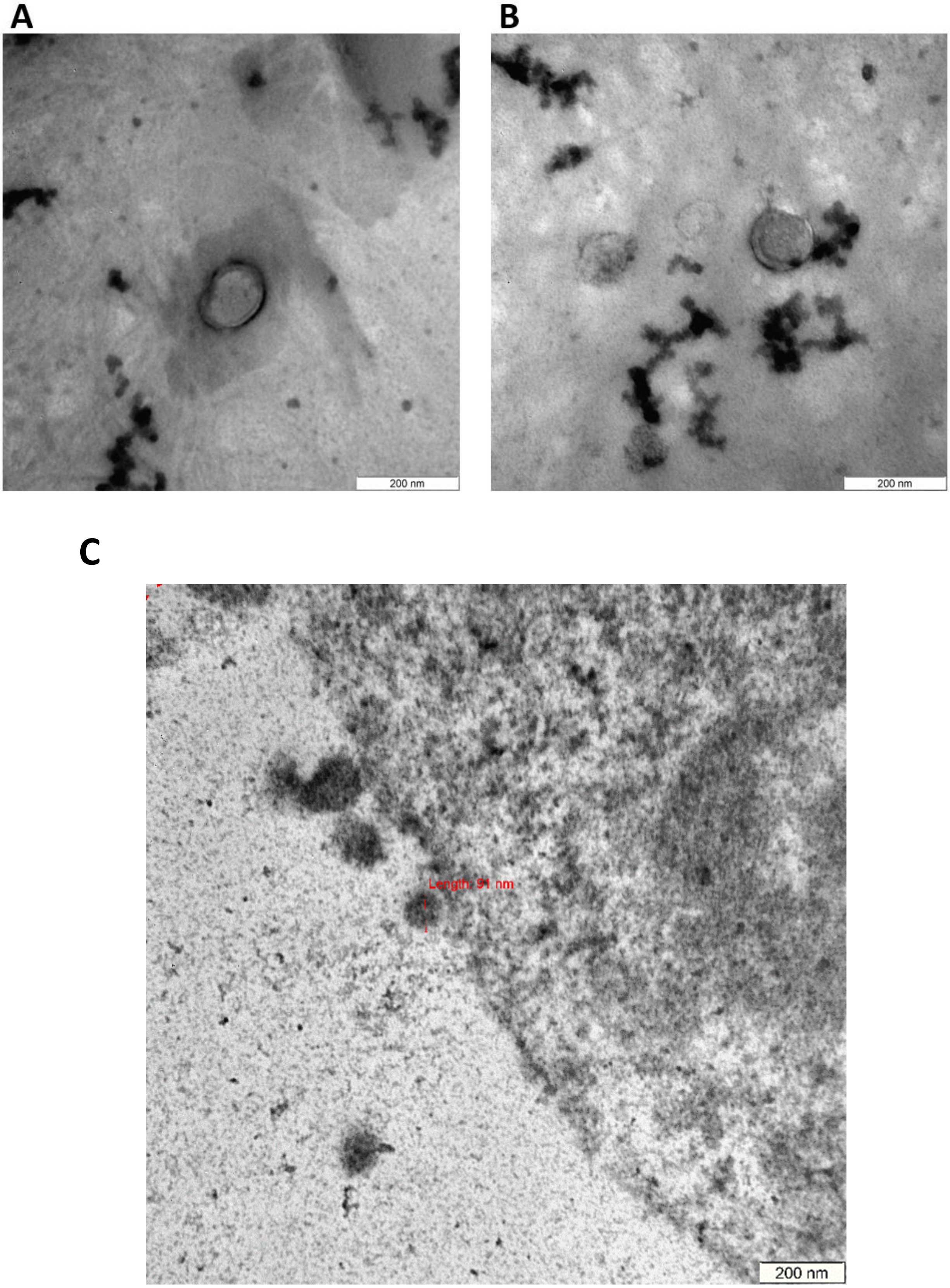

Supplementary Figure S7. Electron microscopic images of (A) purified Virus-like particles (VLP-MmCas12m-TadDE), (B) Lentiviruses, (C) VLP budding.

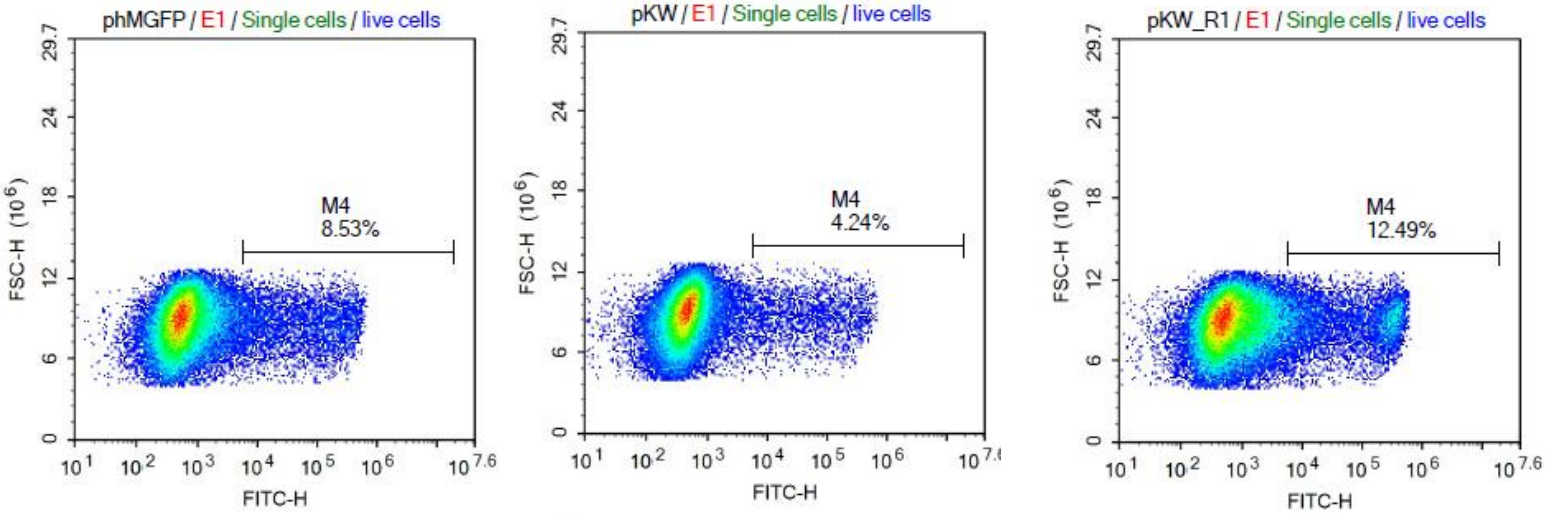

Gate	Count	% live cells	Median X
live cells	46,965	100.00%	578
M4	4,004	8.53%	49,961

Gate	Count	% live cells	Median X
live cells	49,107	100.00%	435
M4	2,080	4.24%	51,821

Gate	Count	% live cells	Median X
live cells	48,806	100.00%	637
M4	6,098	12.49%	64,388

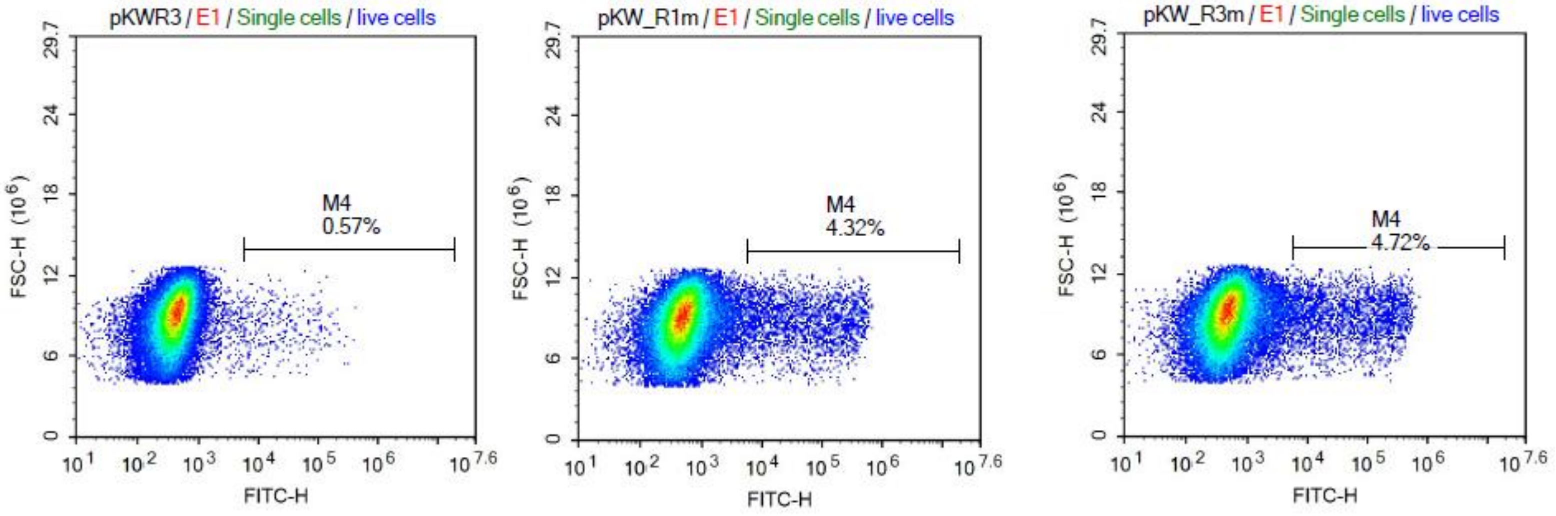

Gate	Count	% live cells	Median X
live cells	49,880	100.00%	398
M4	286	0.57%	20,977

Gate	Count	% live cells	Median X
live cells	48,591	100.00%	476
M4	2,101	4.32%	50,152

Gate	Count	% live cells	Median X
live cells	48,401	100.00%	465
M4	2,284	4.72%	48,188

Supplementary Figure S8: The results of HEK293T transfection with screening plasmids: phMGFP, pKW, pKW_R1, pKW_R3, pKW_R1m, pKW_R3m.

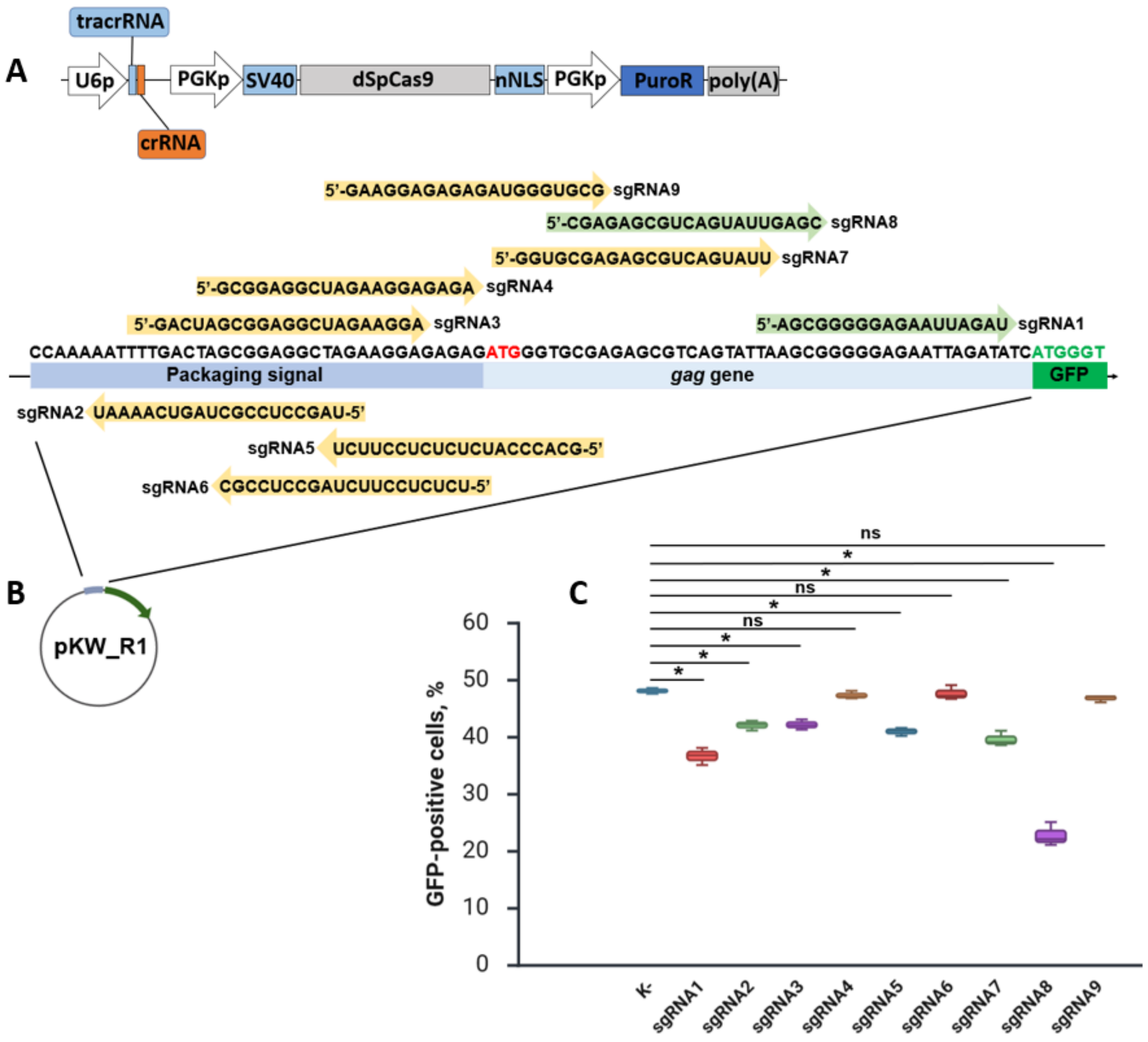

Supplementary Figure S9. Application of the screening system to test dSpCas9 sgRNAs to target HIV-1 DNA. (A) The image of the expression cassette of pdSpCas9-sgRNA used in experiment; (B) The sequence fragment of pKW_R1 plasmid containing HIV-1 DNA; (C) Comparison of the GFP-positive cells proportion during pKW_R1 and pdSpCas9-sgRNAN co-transfection. Detailed description in the text. U6p – the U6 promoter, dSpCas9 – the catalytically inactive *Streptococcus pyogenes* Cas9 gene, SV40 – SV40 large T-antigen nuclear localization signal, nNLS – nucleoplasmin NLS, PuroR – puromycin N-acetyl-transferase gene. (K-) – pKW_R1 and pdSpCas9-sgRNA without sgRNA co-transfected cells. sgRNAs with optimal PAMs (NGG) highlighted in pale green. sgRNAs with non-canonical PAMs highlighted in pale yellow. *gag* start codon highlighted in red. * $p < 0.05$ based on Tukey's test for all comparisons. ns – not significant.

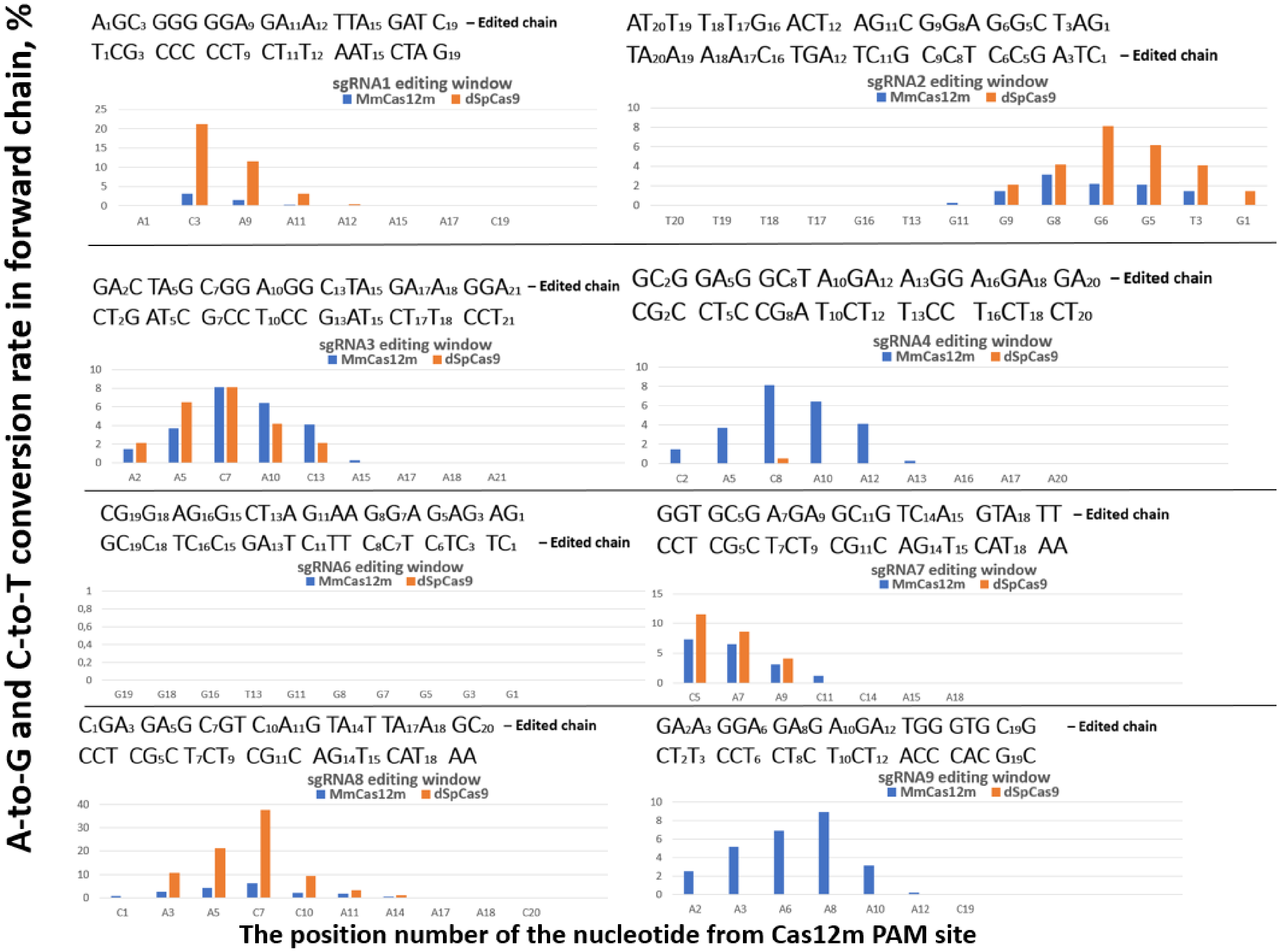

Supplementary Figure S10: The editing efficiency with sgRNA 1-4, 6-9. Editing is mediated by plasmid transfection (pMmCas12m-TadDE-sgRNAN and pdSpCas9-TadDE-sgRNAN).

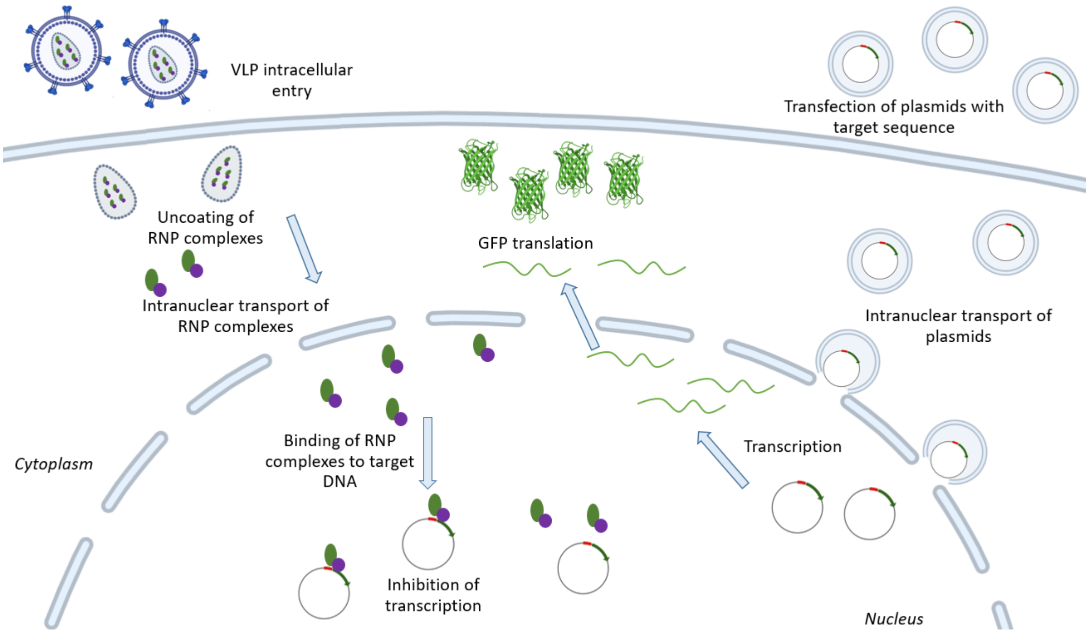

Supplementary Figures S11. The scheme of inhibition of transcription using VLPs. Detailed description in the main text. The scheme of an experiment.

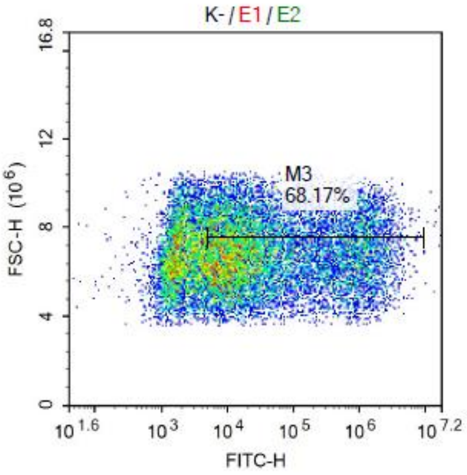

Gate	Count	% E2	Median X
E2	18,506	100.00%	11,916
M3	12,616	68.17%	30,418

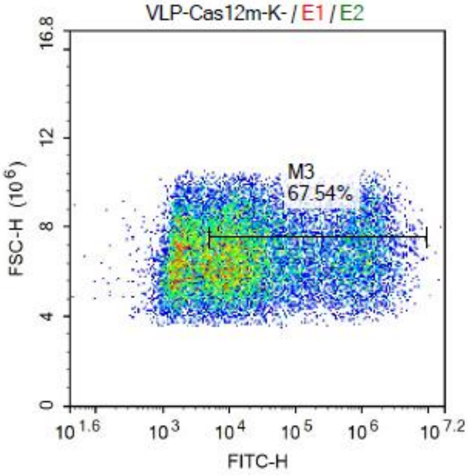

Gate	Count	% E2	Median X
E2	18,704	100.00%	11,739
M3	12,633	67.54%	29,784

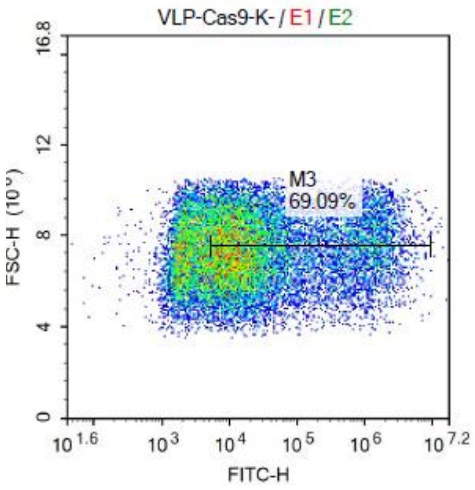

Gate	Count	% E2	Median X
E2	19,493	100.00%	12,383
M3	13,468	69.09%	26,026

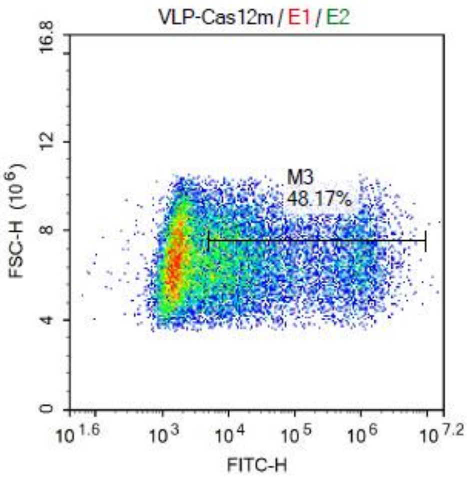

Gate	Count	% E2	Median X
E2	18,705	100.00%	4,558
M3	9,011	48.17%	29,410

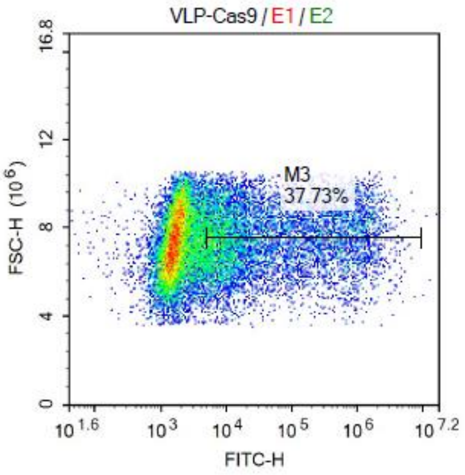

Gate	Count	% E2	Mean X
E2	19,767	100.00%	107,931
M3	7,458	37.73%	276,490

Supplementary Figure S12: Comparison of the GFP-positive cells proportion after transfection with pKW_R1 and the VLPs treatment. (K-) – only transfected cells, (VLP-Cas9-K-, VLP-Cas12m-K-) – cells transfected and treated with VLPs without sgRNA. sgRNA5 is used for VLPs production.

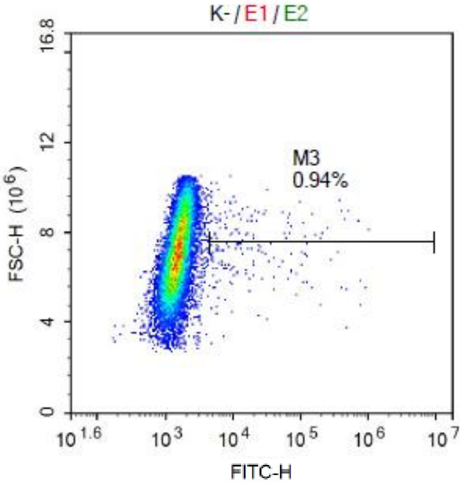

Gate	Count	% E2	Mean X
E2	20,000	100.00%	2,160
M3	187	0.94%	69,559

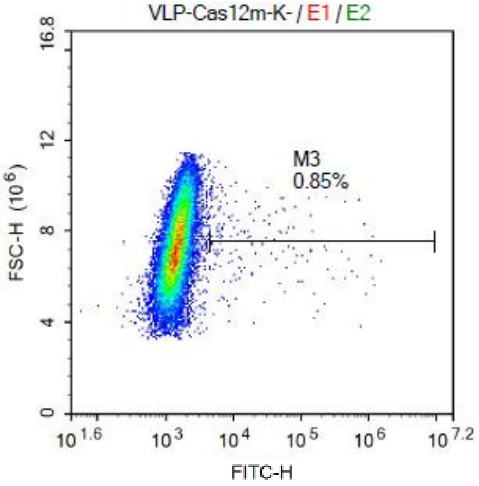

Gate	Count	% E2	Mean X
E2	20,385	100.00%	2,367
M3	173	0.85%	107,800

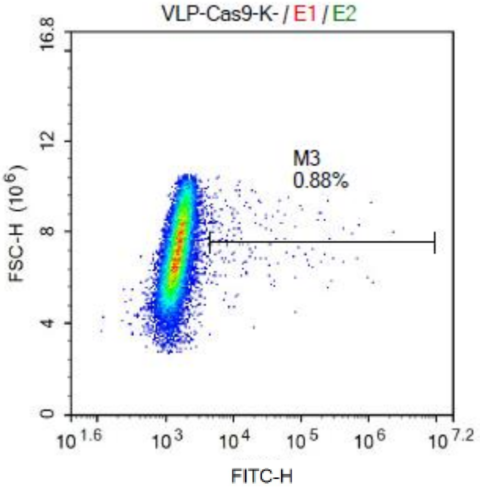

Gate	Count	% E2	Mean X
E2	20,000	100.00%	2,212
M3	175	0.88%	81,893

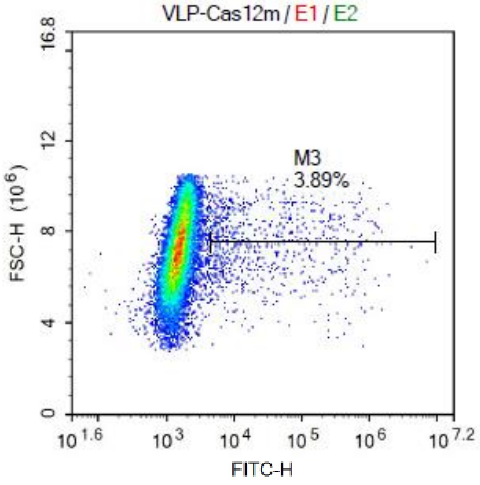

Gate	Count	% E2	Mean X
E2	20,000	100.00%	6,882
M3	778	3.89%	138,810

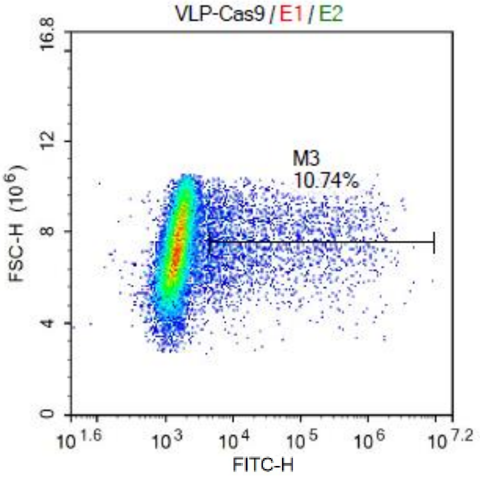

Gate	Count	% E2	Mean X
E2	20,000	100.00%	20,263
M3	2,148	10.74%	175,911

Supplementary Figure S13: Comparison of the GFP-positive cells proportion after transfection with pKW_R3 and the VLPs treatment. (K-) – only transfected cells, (VLP-Cas9-K-, VLP-Cas12m-K-) – cells transfected and treated with VLPs without sgRNA. sgRNA5 is used for VLPs production.

	Nucleotide of the *gag* start codon		
	T8	G7	VLP dose, µL
VLP-Cas12m	1,64; 2,04; 1,92	2,35; 2,21; 2,44	50
VLP-Cas9	3,2; 2,91, 3,54	6,21; 5,93; 6,56	
VLP-Cas12m	2,28; 1,9; 2,45	2,35; 2,21; 2,44	75
VLP-Cas9	4,21; 4,56; 4,32	7,55; 8,01; 7,79	
VLP-Cas12m	1,52; 2,1; 1,87	2,91; 2,65; 2,24	100
VLP-Cas9	5,1; 5,45; 4,95	9,25; 8,72; 9,67	
	Editing efficiency, %		

Supplementary Figure S14: The editing efficiency of start codon nucleotides with sgRNA5. Editing is mediated by the VLPs treatment. Three independent biological replicates.

Description	Spacer	Chromosome	Position	Forward primer	Reverse primer
OT1-Cas12m	tGCAaCCATCTCTCTgCTTCT	8	28341782	GAGGCACTGAAACAAAGCCAG	CCATCATGTGTAGGGCAGGTT
OT2-Cas12m	CtCtCCCATCTCTCTCtTTCT	8	118188067	ATGTGCCAAGAGATGGGGAT	GTAAAGCCAGAGCCCCCAAA
OT3-Cas12m	CtCtCCCATCTCcCTCCTTCT	5	56835631	GAGGTGCGGAATCTTAGGCT	TGTGCCATCTCTTCAGCTTGT
OT4-Cas12m	CtCACtCATCTCTCTgCTTCT	1	20956413	TGTTTTTGTATGGACAGTTAGTCAA	AGTCATTACATCAGCAAGTCCCA
OT1-Cas9	CtCtCCCATCTgTCTCCTTCT	22	36640927	CACTCACTCCTTCCCAGGTC	GGCCAGGCACATTTTCACAAG
OT2-Cas9	tGCAgCCATCTCTCTCCTgCT	9	115090378	GCGTACTCATAGGTTTTGGGGT	CAGCCTGCCACAGGTATGAG
OT3-Cas9	CaCACCCATCcCTCTgCTTCT	18	12362837	ACGTCCTTGCAGAGACATCC	GCCCTGTGTTTGGGAAGGTA
On-target	CGCACCCATCTCTCTCCTTCT				
	Mismatches are highlighted in red				

Supplementary Figure S15: The list of potential off-target sites (in the human genome) during sgRNA5-mediated editing. OT-Cas12m – off-target sites for MmCas12m, OT-Cas9 – off-target sites for dSpCas9.
